# Measurement of Myofilament-Localized Calcium Dynamics in Adult Cardiomyocytes and the Effect of Hypertrophic Cardiomyopathy Mutations

**DOI:** 10.1161/CIRCRESAHA.118.314600

**Published:** 2019-04-11

**Authors:** Alexander J. Sparrow, Kolja Sievert, Suketu Patel, Yu-Fen Chang, Connor N. Broyles, Frances A. Brook, Hugh Watkins, Michael A. Geeves, Charles S. Redwood, Paul Robinson, Matthew J. Daniels

**Affiliations:** 1 From the Division of Cardiovascular Medicine, Radcliffe Department of Medicine (A.J.S., K.S., S.P., Y.-F.C., C.N.B., F.A.B., H.W., C.S.R., P.R., M.J.D.), University of Oxford, United Kingdom; 2 BHF Centre of Research Excellence (A.J.S., S.P., Y.-F.C., C.N.B., F.A.B., H.W., C.S.R., P.R., M.J.D.), University of Oxford, United Kingdom; 3 BHF Centre of Regenerative Medicine (M.J.D.), University of Oxford, United Kingdom; 4 Department of Cardiology, Oxford University NHS Hospitals Trust, United Kingdom (H.W., M.J.D.); 5 Department of Biosciences, University of Kent, Canterbury, United Kingdom (M.A.G.); 6 Department of Biotechnology, Graduate School of Engineering, Osaka University, Suita, Japan (M.J.D.).

**Keywords:** calcium, cardiomyopathies, fluorescence, mutation, sarcomere

## Abstract

**Rationale:**

Subcellular Ca^2+^ indicators have yet to be developed for the myofilament where disease mutation or small molecules may alter contractility through myofilament Ca^2+^ sensitivity. Here, we develop and characterize genetically encoded Ca^2+^ indicators restricted to the myofilament to directly visualize Ca^2+^ changes in the sarcomere.

**Objective:**

To produce and validate myofilament-restricted Ca^2+^ imaging probes in an adenoviral transduction adult cardiomyocyte model using drugs that alter myofilament function (MYK-461, omecamtiv mecarbil, and levosimendan) or following cotransduction of 2 established hypertrophic cardiomyopathy disease-causing mutants (cTnT [Troponin T] R92Q and cTnI [Troponin I] R145G) that alter myofilament Ca^2+^ handling.

**Methods and Results:**

When expressed in adult ventricular cardiomyocytes RGECO-TnT (Troponin T)/TnI (Troponin I) sensors localize correctly to the sarcomere without contractile impairment. Both sensors report cyclical changes in fluorescence in paced cardiomyocytes with reduced Ca^2+^ on and increased Ca^2+^ off rates compared with unconjugated RGECO. RGECO-TnT/TnI revealed changes to localized Ca^2+^ handling conferred by MYK-461 and levosimendan, including an increase in Ca^2+^ binding rates with both levosimendan and MYK-461 not detected by an unrestricted protein sensor. Coadenoviral transduction of RGECO-TnT/TnI with hypertrophic cardiomyopathy causing thin filament mutants showed that the mutations increase myofilament [Ca^2+^] in systole, lengthen time to peak systolic [Ca^2+^], and delay [Ca^2+^] release. This contrasts with the effect of the same mutations on cytoplasmic Ca^2+^, when measured using unrestricted RGECO where changes to peak systolic Ca^2+^ are inconsistent between the 2 mutations. These data contrast with previous findings using chemical dyes that show no alteration of [Ca^2+^] transient amplitude or time to peak Ca^2+^.

**Conclusions:**

RGECO-TnT/TnI are functionally equivalent. They visualize Ca^2+^ within the myofilament and reveal unrecognized aspects of small molecule and disease-associated mutations in living cells.

## Abstract 2

Supplemental Digital Content is available in the text.

The myofilament converts the chemical energy of ATP into mechanical energy, a process regulated by Ca^2+^. Mutations in myofilament components cause common (1:500) inherited conditions like hypertrophic cardiomyopathy (HCM).^1–3^ HCM causing mutations produce hypercontractility, energetic compromise,^4–6^ and change Ca^2+^ utilization within the cardiomyocyte. Altered intracellular Ca^2+^ may be directly proarrhythmic, causing sudden death, and activate hypertrophic signaling pathways that produce the cardiac hypertrophy, and premature heart failure, observed in HCM patients. Interestingly, increased myofilament Ca^2+^ affinity arises from different mutations.^7,8^ Prevalent thick filament mutations increase the Ca^2+^ sensitivity of contractility by altering actomyosin affinity and modifying conformational changes in the myosin motor domain.^9^ Conversely, thin filament mutations in the regulatory components TnT (troponin T) and I (TnI) increase the affinity of TnC (troponin C) for Ca^2+^. Some consequences of trapping Ca^2+^ in the sarcomere by the introduction of Ca^2+^ sensitizing HCM mutations are known.^10–12^ Briefly, raised myofilament Ca^2+^ binding produces secondary alterations in cytoplasmic and sarcoplasmic reticulum [Ca^2+^] in addition to altered activation/regulation of key Ca^2+^ handling proteins (eg, ryanodine receptor, sarco-endoplasmic reticulum ATPase 2a, and phospholamban). Hypertrophic signaling via calcineurin/NFAT (nuclear factor of activated T-cells) and ERK (extracellular signal-regulated kinase) promotes cellular hypertrophy.^13^ These factors, combined with altered mechanosensing^14^ and energetics,^2,15^ drive the stereotyped end-organ changes seen in patients.

**Editorial, see p 1151**

**Meet the First Author, see p 1142**

Ca^2+^ cycling changes in HCM are typically assessed using chemical Ca^2+^ dyes.^10,11,13^ However, dyes are not spatially restricted^16–19^ and thus report whole-cell phenomena. The patch clamp technique^20^ can resolve subcellular Ca^2+^ events^10,11^ but is technically challenging and low throughput. Genetically encoded calcium indicators (GECIs) offer an alternative strategy for Ca^2+^ assessment^17,19^ and may be restricted to subcellular compartments by a defined sequence tag or protein fusion. Myofilament specific calcium probes remain elusive, in part, because of the concern that the bulky 30×40×70 Å adducts of the smallest GECIs^21^ are unlikely to be tolerated by the paracrystalline sarcomeric environment^22,23^ leading to perturbed function of the indicator, the fusion partner, or the cell. However, fluorescence resonance energy transfer-based cAMP indicators conjugated to myofilament proteins that do not affect cardiomyocyte contractility have been reported.^24^

GECIs are available with different calcium sensor domains, tuned to different affinities, in a range of spectral hues. The CaM (calmodulin)/M13 calcium sensor domain appears in most of the indicators successfully reported in cardiomyocytes but not all GECIs work in all cardiac models.^17^ For example, tools improved for neuronal applications may lose functionality in cardiac cells,^17^ for example, TN-XL^25^ and TN-XXL.^26^ Subcellular targeting of GECIs within cardiomyocytes to probe Ca^2+^ in the sarcoplasmic reticulum^27^ and, by conjugation of GCaMP2.2 to FKBP12.6, Ca^2+^ efflux from the sarcoplasmic reticulum^28^ has been demonstrated. These probes visualize the Ca^2+^ store, but currently no equivalent tool to study Ca^2+^ at the myofilament, where Ca^2+^ regulates contraction exists.

Because contractility underpins cardiac function, there is interest in development of small molecule modulators of myofilament contractility.^22,29–31^ Importantly, these agents should not affect Ca^2+^ handling, which may be proarrhythmic. Application of unrestricted, or myofilament localized, indicators may give greater mechanistic insight into this subclass of drugs, which include the myosin ATPase inhibitor MYK-461,^30^ a novel therapy for HCM, and levosimendan^31^ a myofilament activator developed for heart failure.

Here, we produce and characterize myofilament localized Ca^2+^ sensors to directly visualize changes in Ca^2+^ flux at the myofilament occurring in response to small molecules or disease-causing mutations. We reveal how 2 well-characterized thin filament HCM causing mutations in cTnT (R92Q)^4,10,32,33^ and cTnI (R145G)^10,34–36^ are from a Ca^2+^ handling perspective mechanistically similar at the myofilament but distinct in the cytoplasm. The combination of probes reveals biology previously inferred. The method increase scale, reduces animal use, and is compatible with existing imaging infrastructure and single cell protocols.

## Methods

The software, the plasmids, or the data that support the findings of this study are available from the corresponding author on reasonable request.

### Isolation of Guinea Pig Left Ventricular Cardiomyocytes

This investigation was approved by the Animal Welfare and Ethical Review Board at the University of Oxford and conforms to the UK Animals (Scientific Procedures) Act, 1986, incorporating Directive 2010/63/EU of the European Parliament. As described,^10^ left ventricular cardiomyocytes were isolated from male (to prevent confounding effects of hormonal cycle) adolescent (400 g) guinea pig hearts by collagenase perfusion for 5 minutes then placed on a shaker for a further 10 minutes. Left ventricular cardiomyocytes (1.5×10^5^ cells per mL) were incubated in ACCITT_3_ culture medium^37^ at 37°C and 5% CO_2_.

### Adenoviral Transduction

RGECO-TnT (plus BamHI linker) and RGECO-TnI (plus XhoI linker) were cloned into a shuttle vector. RGECO and RGECO-TnI adenoviruses were generated, purified, and titred by Welgen, Inc (Worcester, MA). RGECO-TnT, wild-type (WT) cTnT, cTnT R92Q, WT cTnI, and cTnI R145G adenoviruses were generated as described.^10^ Immediately after isolation Guinea pig cardiomyocytes (GPCMs) were adenovirally cotransduced for 48 hours with RGECO (multiplicity of infection=444) and either WT cTnT (795), cTnT R92Q (933), WT cTnI (795), or cTnI R145G (795). Cotransductions of RGECO-TnT (multiplicity of infection=795) were with either WT cTnI (795) or cTnI R145G (1590). Cotransductions of RGECO-TnI (multiplicity of infection=444) were with either WT cTnT (795) or cTnT R92Q (1493).

### Protein Purification

N- and C-terminal eGFP (enhanced green fluorescent protein) conjugates of human recombinant TnT, TnI, and TnC were cloned into pMW172 vector using 3′ NdeI and 5′ EcoRI restriction sites and an internal HindIII restriction site linker. RGECO and RGECO-TnT were similarly subcloned into pMW172. Proteins were expressed in BL21-DE3(pLysS) *Escherichia coli* induced using 0.4 mmol/L Isopropyl β-D-1-thiogalactopyranoside for 4 hours. Bacterial pellets recovered by centrifugation (10 000×*g* for 10 minutes) were lysed in buffer containing 25 mmol/L Tris-HCl, pH 7.5, 20% sucrose, 1 mmol/L EDTA, 200 mmol/L NaCl,5 mol/L urea, and 0.1% Triton X-100. All proteins were purified using an AKTA-UPC900 FPLC using HiTrap FF chromatography columns (GE Healthcare, Amersham). GFP (green fluorescent protein)-TnT and TnT-GFP were purified using sequential cation (in buffer containing 6mol/L urea, 1 mmol/L EDTA, 1 mmol/L 2-mercaptoethanol, 20 mmol/L MOPS, pH 6.0) followed by anion (in buffer containing 6mol/L urea, 1 mmol/L EDTA, 1 mmol/L 2-mercaptoethanol 50 mmol/L Tris-HCl pH 8.0) exchange chromatography. GFP-TnC and TnC-GFP were purified by anion (pH 8.5) exchange chromatography. GFP-TnI and TnI-GFP were purified by sequential cation and anion exchange chromatography. RGECO and RGECO-TnT were purified using sequential anion (pH 8.0) exchange chromatography, ammonium sulphate fractionation (to 50% for RGECO and 35% for RGECO-TnT) and finally hydrophobic interaction chromatography (in buffer containing 30% ammonium sulphate, 200 mmol/L NaCl_2_, 1 mmol/L ditiothretol, and 30 mmol/L 4-(2-hydroxyethyl)-1-piperazineethanesulphonic acid [HEPES]). Ion exchange columns were eluted using a 0 to 2 mol/L NaCl gradient, the hydrophobic interaction chromatography column was eluted with a 30% to 0% ammonium sulphate gradient. Six-Histidine-tagged RGECO-TnI was cloned into a PET23a vector and expressed as above and purified using a HisTrap HP column (GE Healthcare) in a buffer containing 15.48 mmol/L Na_2_HPO_4_, 4.52 mmol/L NaH_2_PO_4_, 500 mmol/L NaCl, and 6mol/L Urea (Histag buffer), followed by washing in Histag buffer with 20 mmol/L Imidazole (pH 7.0), then eluted in an increasing Histag buffer gradient containing 500 mmol/L Imidazole (pH 7.0). Eluted fractions were assessed for purity using 12% SDS-PAGE gels, stained with Coomassie brilliant blue. Histag was cleaved using a thrombin kit (Merck) following the manufacturer’s instructions. WT human recombinant TnT, TnI, TnC, and Ala-Ser-α-TM^38^ were purified as previously described.^39^ Troponin complex containing either WT, GFP-TnT, TnT-GFP, GFP-TnC, TnC-GFP, GFP-TnI, or TnI-GFP were reconstituted by dialysis into buffer containing 10 mmol/L imidazole pH 7.0, 1 mmol/L DTT, 0.01% azide, 0.1 mol/L CaCl_2_, 6 mol/L urea, and 1 mol/L KCl. Urea was reduced stepwise from 6 to 2 then 0 mol/L, then KCl was reduced stepwise from 1 mol/L to 800 to 600 to 400 to 200 mmol/L KCl in a series of 3-hour dialyzes. Tn complex was purified using size exclusion chromatography in 200 mmol/L KCl dialysis buffer. Purity was analyzed by SDS-PAGE. Purified troponin complexes were dialyzed into buffer containing 5 mmol/L 1,4-piperazinediethanesulphate pH 7.0, 3.87 mmol/L MgCl_2_, 1 mmol/L DTT for ATPase assay experiments. Actin and myosin S1 were extracted from rabbit skeletal muscle as described.^38,40^

### In Vitro Actomyosin ATPase Assays

ATPase assays were performed as described.^8,39^ Briefly, a master stock of 3.5 μmol/L actin, 0.5 μmol/L myosin S1, 0.5 μmol/L Ala-Ser-α-TM, and 0.5 μmol/L Tn complex was mixed in buffer containing 5 mmol/L 1,4-Piperazinediethanesulphate pH 7.0, 3.87 mmol/L MgCl_2_, and 1 mmol/L DTT. To ensure precise thin filament protein stoichiometry, each stock was centrifuged at 384 000×*g* for 15 minutes. Recovered pellets were aliquoted and set to a range of [Ca^2+^]_free_ from pCa 4.5 to 8.5 using Maxchelatior (http://maxchelator.stanford.edu/CaEGTA-TS.htm). ATPase reactions were initiated by addition of 3 mmol/L ATP and incubated at 37°C for 15 minutes. Each reaction was quenched in 5% TCA, finally, 1% ammonium molybdate in 0.5 mol/L H_2_SO_4_, followed by 40% iron(II)sulphate in 0.5 mol/L H_2_SO_4_ was used to measure inorganic phosphate. Absorbance (A_700_) measurements were converted to absolute activity (s^−1^). Calcium-sensitivity data were fitted to the Hill equation using KaleidaGraph (Synergy Software).


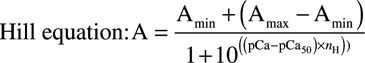


Where: A=ATPase rate; A_min_=Minimum ATPase rate; A_max_=Maximum ATPase rate; pCa=−log [Ca^2+^]; pCa_50_=−log [Ca^2+^] required for half maximum ATPase activity; *n*_H_=Hill coefficient.

### Calcium Binding K_d_ Calculations

For steady-state Ca^2+^ binding affinity (*K*_d_) for RGECO, RGECO-TnI, and RGECO-TnT, 3 μmol/L of protein was dialyzed into buffer containing 130 mmol/L NaCl, 10 mmol/L HEPES, 1 mmol/L dithiothretol, 1.3 mmol/L MgCl_2_, pH 7.3. A range of _free_[Ca^2+^] conditions was set between 3.16 nmol/L (pCa 8.5) and 31.6 μmol/L (pCa 4.5) using 1 mmol/L EGTA and the corresponding concentration of CaCl_2_. Steady-state fluorescence readings were made in an Ultraclear bottom 96-well microplate using a FLUOstar Omega plate reader (BMG LABTEC, Germany), using 544 nm excitation and 590/10 nm emission filters at both 25°C and 37°C. Fluorescence emission intensities were plotted versus _free_[Ca^2+^] and fitted to the Hill equation to calculate *K*_d_ values for each protein and condition. For Ca^2+^ displacement (*k*_off_), 125 nmol/L RGECO-TnT plus 5 µmol/L CaCl_2_ was mixed with 5 mmol/L EGTA in a buffer containing 130 mmol/L NaCl, 10 mmol/L HEPES 1.3 mmol/L MgCl_2_, 1 mmol/L dithiothreitol, pH 7.3 with NaOH. RGECO or RGECO-TnT was loaded into a stopped-flow system (HiTech Scientific, Bradford-on-Avon, United Kingdom), concentrations after mixing 1:1 in the stopped-flow. Fluorescence was excited at 546 nm (100 W Xe/Hg lamp and monochromator) and emission monitored through an OG-590 glass filter. Data were fitted to a single exponential decrease in fluorescence of 50% to 100%, depending on temperature. For calcium binding (*k*_on_), 125 nmol/L RGECO-TnT was measured at 10 µmol/L free calcium by mixing with a buffer containing 2.125 mmol/L Ca. EGTA and 0.2 mmol/L EGTA with the same filter set as before. Fluorescence increase on Ca^2+^ addition was fitted to a single exponential. The observed single exponential rate constant, *k*_obs_, was extracted for *k*_on_ and *k*_off_ for both sensors, at a range of temperatures between 25°C and 37°C. To estimate the dissociation constant, we using the equations *K*_d_=*k*_obs_−off [Ca]/ *k*_obs_ on; these were comparable to steady-state *K*_d_ values.

### Determination of Quantum Yield and Molar Extinction Coefficient

Quantum yield and molar extinction coefficient were determined for each Ca^2+^ sensor following.^41^ Quantum yield standards were mCherry (for RGECO) and RGECO for RGECO-TnT. The concentration of protein in buffer containing 10 mmol/L HEPES, 1.3 mmol/L MgCl_2_, and 1 mmol/L dithiothreitol was determined using BCA assay (Pierce) and set to either pCa 4.5 of 8.5.The protein concentration was reduced to an absorbance at the excitation wavelength between 0.2 and 0.6. Dilutions to absorbance’s of 0.01, 0.02, 0.03, and 0.04 were made for each protein solution and standard. The fluorescence emission spectra at Ex F_568_ were recorded using an RF-1501 spectrofluorimeter (Shimadzu, Japan). Total fluorescence intensities were obtained by integration. Integrated fluorescence intensity versus absorbance was plotted for each protein and each standard. Quantum yields were determined from the slopes (S) of each line using the equation: Φprotein = Φstandard×(S_protein_/S_standard_). Extinction coefficients were determined from the absorption spectrum of both RGECO and RGECO-TnT at pCa 8.5 and 4.5 in the buffer above using a plate reader (BMG LABTECH, Germany). Peak absorbance wavelengths were determined to be A_465_ in low Ca^2+^ and A_581_ in high Ca^2+^. Absorbance measurements were converted to 1 cm path length using BMG Omega software. Extinction coefficients were calculated by dividing the peak absorbance maximum by protein concentration.

### Immunofluorescence

GPCMs were fixed in 4% paraformaldehyde, washed in PBS, permeabilized and blocked in 0.1% Triton X-100, 3% BSA in PBS. Primary antibodies (1:500 anti-α actinin [Sigma, clone EA-53, A7811], 1:200 anti-DsRed [Clontech, 632496], 1:8 anti-FLAG-tag [Sigma, F1804]) were added overnight at 4°C, washed in PBS, and secondary antibodies added (goat anti-rabbit IgG Alexa 568 [1:200; Invitrogen, A21069] and goat anti-mouse IgG Alexa 633 [1:200; Invitrogen, A21053]) for 1 hour at room temperature, washed in PBS and mounted on slides in ProLong Diamond antifade with DAPI (ThermoFisher). Slides were imaged on a Leica TCS SP5 confocal microscope with a ×63 oil immersion lens, images data were extracted with Leica LAS and ImageJ (National Institutes of Health). Control slides were prepared excluding the primary antibody for all IF experiments, negligible background fluorescence was detected in all cases.

### Subcellular Fractionation and Western Blotting

GPCMs were fractionated to recover cytoplasmic and sarcomeric/cytoskeletal samples following.^42^ A total of 300 000 cardiomyocytes were centrifuged, resuspended in buffer containing, 20 mmol/L Tris-HCl pH 7.4, 2 mmol/L EDTA, 0.5 mmol/L EGTA, 0.3 mmol/L sucrose, and homogenized for 1 minute. Cells were pelleted at 1000×*g* for 5 minutes, the supernatant/cytoplasmic fraction was retained for SDS-PAGE analysis and Western blotting. The cell pellet was washed 3 times in fractionation buffer + 1% Triton X-100 each time pelleting the sarcomeric fraction by centrifugation at 1000×*g* for 5 minutes. Finally, the sarcomeric fraction was resuspended in fractionation buffer (without Triton X-100) and prepared for Western blotting. Subcellular fractions and GPCM lysates were run on a 12% polyacrylamide gel, transferred onto PVDF (polyvinylidene difluoride) membrane, and blocked with 5% bovine milk in Tris-buffered saline, 0.1% Tween-20 (TBS-T) for 1 hour. Primary antibodies were incubated overnight at 4°C in 5% bovine milk in TBS-T (1:2000 anti-cTnT [Sigma, SAB2108239], 1:4000 anti-ERK [Cell Signaling Technologies, 4695s], 1:5000 anti-FLAG-tag [Sigma, F3165], 1:10 000 anti-GAPDH [Millipore, ABS16], and 1:2000 anti-TnI [TNNI3, Aviva Systems Biology, ARP41391_P050]). Membranes washed in TBS-T were incubated in secondary antibodies (1:10 000 anti-rabbit HRP [GE Healthcare, NA934], 1:10 000 anti-mouse HRP [GE Healthcare, NA931]) for 1 hour at room temperature, washed in TBS-T then developed with ECL select (GE Healthcare) and imaged on a ChemiDoc MP (BioRad). Densitometric measurements to compare single versus double infected levels of FLAG-tag protein were performed using Image Lab software (BioRad) and normalized to GAPDH loading controls for each comparison.

### Sarcomere Length Measurements

Sarcomere length measurements were performed using IonOptix µstep apparatus and the manufacturers’ standard operating instructions. Cells, electrically paced at 40 volts, 0.5 Hz, 37°C were perfused with buffer containing 150 mmol/L NaCl, 10 mmol/L HEPES, 7 mmol/L glucose, 1 mmol/L MgCl_2_, 1 mmol/L KCl, 0.3 mmol/L NaH_2_PO_3_, and 1.8 mmol/L CaCl_2_, at pH 7.4. Cells are displaying asynchronous contractility, excessive blebbing/dysmorphology were ignored for acquisition. Similarly, cells with contractile magnitudes or velocities exceeding 2 SDs from the mean on analysis were also excluded because of phenotypic heterogeneity arising in cultured primary cells.

### Imaging and Analysis

GPCMs imaged on an Olympus IX81 inverted microscope (Olympus, Japan) with a C-1900 EMCCD camera (Hamamatsu, Japan) were electrically paced at 0.5 Hz in a humidified chamber at 37°C. Videos (28 seconds, 25 fps) were acquired through an Olympus UPlanFLN 10× lens (NA 0.3) with the RFP filter set of 560/25 nm (excitation), 620/60 nm (emission) with a 565 nm dichroic mirror using CellR software (Olympus) as previously described.^43^ RGECO, RGECO-TnI, and RGECO-TnT adenovirally transduced GPCMs were incubated with 250 nmol/L MYK-461, 200 µmol/L omecamtiv mecarbil or 10 µmol/L levosimendan 20 minutes before imaging. For Fluo-4 comparison, RGECO-TnT transduced cardiomyocytes were loaded with 1 μmol/L Fluo-4 as per the manufacturer’s instructions for 5 minutes and imaged using RFP and GFP (485/20–25 nm [excitation], 525/50 nm [emission] with a 495 nm dichroic mirror) filter sets.

Raw image data were extracted using CellR (Olympus) and analyzed in Excel (Microsoft). From a single cell, 10 transients were extracted and averaged to give a single transient in a 2-second interval per cell. Time to 50% (T_50_) contraction, T_50_ baseline (from peak), time of peak, or time above 50% was determined. Peak intensity analysis was performed as previously described for intensiometric sensors.^44,45^


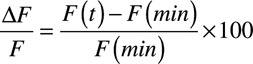


Where F(t) is the fluorescence intensity at time (t), and F(min) the minimum fluorescence intensity during the averaged transient.

### Statistics

For all single cells experiments, comparisons were from at least 3 separate cell isolations, where approximately equal numbers of cells (±5) were taken from each preparation on an experiment-to-experiment basis. Data were first tested for normality (D’Agostino-Pearson). Sarcomere length measurements were made using unpaired Student *t* test. Wilcoxon *T* test was used for the comparison of Fluo-4 to RGECO-TnT. RGECO to RGECO-TnT/TnI and pacing frequencies were compared using nonparametric Kruskal-Wallis 1-way ANOVA. Drugs and WT to mutant troponin were compared using nonparametric Mann-Whitney test (Graphpad Prism). Once acquired, all data were blinded before extraction and analysis.

## Results

### Development and In Vitro Characterization of Myofilament RGECO Ca^2+^ Sensors

**Figure 1.**
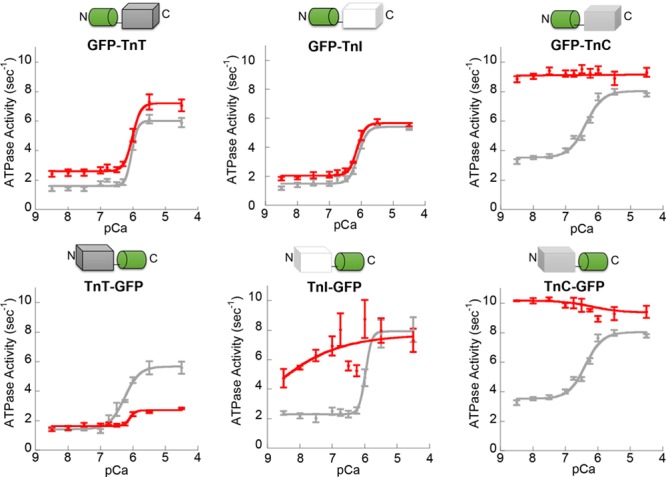
**The effect of GFP (green fluorescent protein) conjugation to the N and C terminus of troponin subunits on myofilament function.** Myofilament function was assessed using in vitro actin-activated actomyosin S1 ATPase assays. Control (unconjugated) troponin complexes (gray lines) were compared pairwise to troponin complex reconstituted with subunits conjugated to the N or the C terminus of GFP as illustrated (red lines). n=3–5 error bars are±SEM. TnC indicates Troponin C; TnI, Troponin I; and TnT, Troponin T.

**Figure 2.**
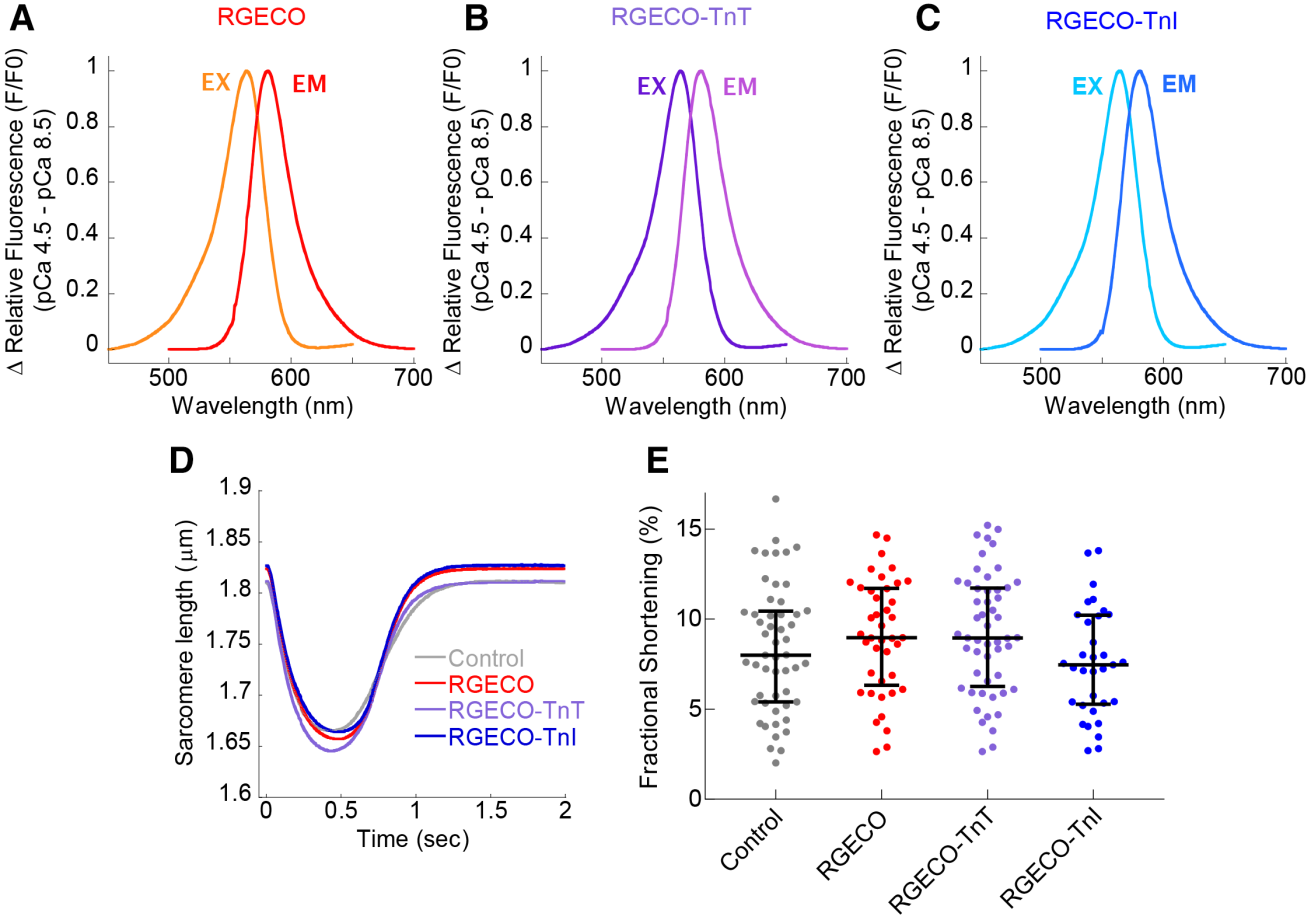
**Fluorescent and contractile properties of RGECO, RGECO-TnT (Troponin T), and RGECO-TnI (Troponin I**). Steady-state fluorescence excitation, emission spectra (peak excitation [Ex] =564 nm)/peak emission (Em) =581 nm) were obtained at pCa4.5 and subtracted from paired spectra at pCa8.5 for purified recombinant RGECO (**A**), RGECO-TnT (**B**), and RGECO-TnI (**C**). Sarcomere shortening during electrical pacing (0.5 Hz) of isolated adult cardiomyocytes was used to test cardiac contractile function in nontransduced and either RGECO, RGECO-TnT, or RGECO-TnI transduced Guinea pig cardiomyocytes (GPCMs) by measurement of the sarcomeric length during contraction (n=33–52; **D**) or by fractional shortening (**E**).

To develop a myofilament-restricted GECI, fusion proteins of GFP at the N and C terminus of each troponin subunit were screened to identify sites preserving in vitro actomyosin ATPase Ca^2+^ dependent regulation. GFP conjugation to the N terminus of either TnT or TnI maintained Ca^2+^ regulation (Figure 1; Online Table I). Subsequently, the red GECI RGECO^41^ was conjugated to the N terminus of TnT and TnI (Online Figures X and XI). In vitro characterization of the calcium indicator properties of RGECO-TnT and RGECO-TnI showed *K*_d_ of 764.5±17.6 nmol/L and 657.3±27.3 nmol/L, respectively, under physiological pH,^46^ temperature and magnesium concentration^47^ (Online Figure I), with preservation of dynamic range and excitation/emission spectra (Figure 2A through 2C; Online Table II). This is compatible with previous reports^41^ (RGECO *K*_d_ =484 nmol/L at 25°C and 3 mmol/L MgCl_2_). At physiological temperature, Ca^2+^ on (*k*_obs_ 16.7 μmol/L^−1^s^−1^ at 10 μmol/L free Ca^2+^) and off (*k*_off_ =16.9 s^−1^) rates for myofilament RGECO were approximately twice those observed at 25°C (Online Figure II). Importantly, in intact, but unloaded, GPCMs the presence of RGECO-TnT/TnI did not affect sarcomere shortening compared with uninfected controls (Figure 2D and 2E; Online Figure III).

### Characterization of RGECO-TnT and RGECO-TnI Ca^2+^ Sensors in Cardiomyocytes

**Figure 3.**
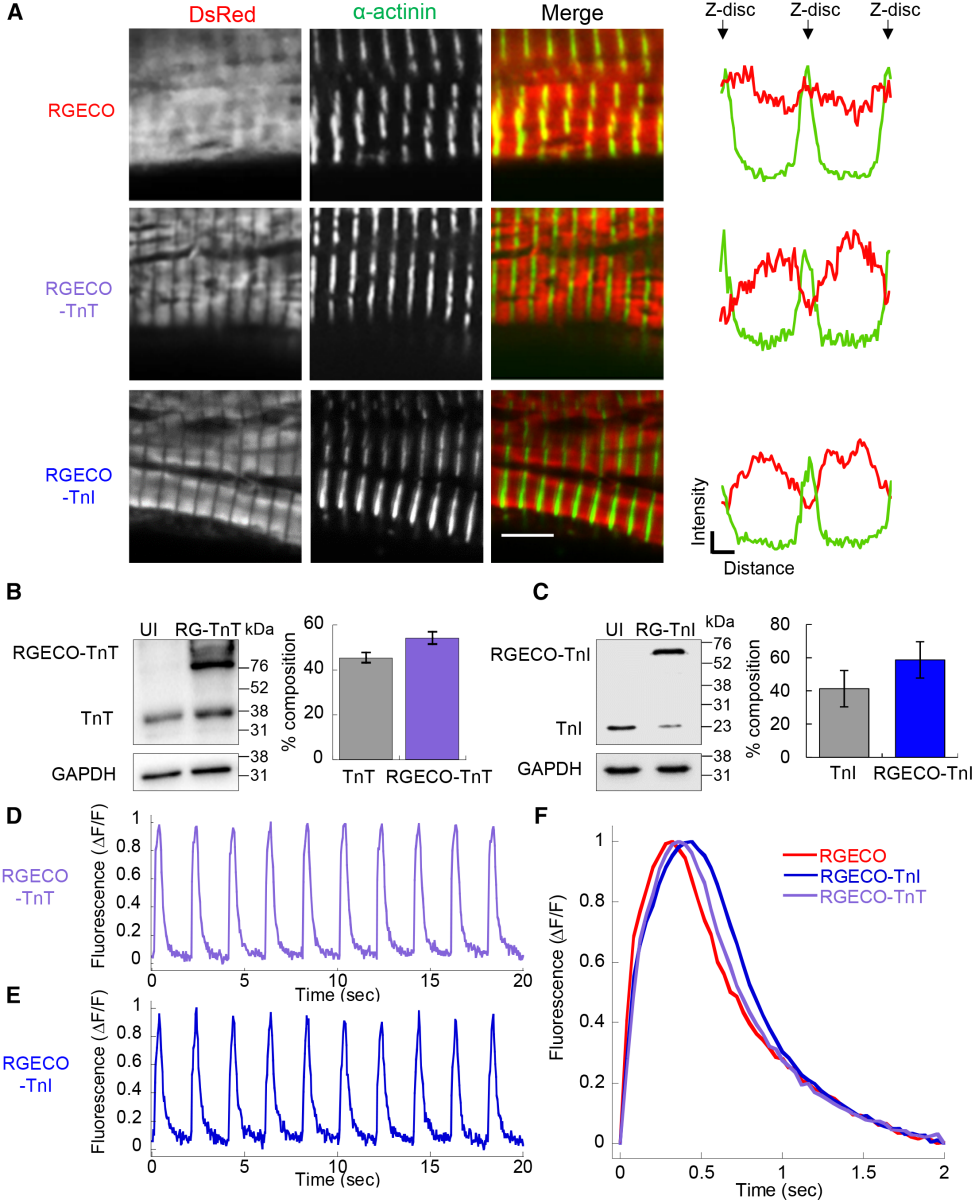
**Characterization of RGECO, RGECO-TnT (Troponin T), and RGECO-TnI (Troponin I) in Guinea pig cardiomyocytes (GPCMs).** Adenovirally expressed RGECO-TnT and RGECO-TnI (red in the merged images) localizes to the I band in confocal microscopy of GPCMs, while RGECO shows diffuse staining (n=4; **A**); Z-disks are revealed by α-actinin staining (green in the merged images). Scale bar =5 μm. Intensity profile plots spanning 2 sarcomeres, α-actinin (green line) labels the z-disc, DsRed (red) labels the Ca^2+^ sensors. Western blot analysis of GPCMs transduced with RGECO-TnT (**B**) or RGECO-TnI (**C**) indicates that 54.3±5.5% (n=5)/58.7±11.0% (n=4), respectively, of Troponin is composed of RGECO conjugated Troponin. Raw Ca^2+^ transients of paced GPCMs transduced with RGECO-TnT (**D**) or RGECO-TnI (**E**). Averaged Ca^2+^ transients of 0.5 Hz paced GPCMs transduced with RGECO, RGECO-TnT, or RGECO-TnI (**F**) was used to compare Ca^2+^ transients between cytoplasmic RGECO and myofilament specific RGECO-TnT and RGECO-TnI.

RGECO-TnT or RGECO-TnI localize to the sarcomere, without Z-disk accumulation, following adenoviral transduction of GPCMs. Conversely, unconjugated RGECO is broadly distributed with weak Z-disk accumulation (Figure 3A; Online Figure IV). Forty-eight hours following viral infection, total TnI contained 58.7±11.0% (n=5) RGECO-TnI and similarly TnT contained 54.3±5.5% (n=4) RGECO-TnT (Figure 3B and 3C). RGECO-TnI and RGECO-TnT both show cyclical fluorescence in paced cardiomyocytes (Figure 3D and 3E), with equivalent kinetics in untreated GPCMs stimulated at 0.5 Hz (Figure 3F; Online Table III). The on and off rates of fluorescence differ in both myofilament localized indicators compared with the unrestricted RGECO. On rates were significantly slower (ΔT_50_ on =47±5 and 39±4 ms for RGECO-TnT and RGECO-TnI, respectively), while off rates were significantly faster (ΔT_50_ off =−26±8 and −41±11 ms for RGECO-TnT and RGECO-TnI, respectively) than unrestricted RGECO (Online Table III). Interestingly, these rate differences are qualitatively preserved when RGECO-TnT emissions are compared with the chemical dye Fluo-4 (Online Figure V).

To explore the sensitivity of RGECO-TnI/RGECO-TnT to detect changes in Ca^2+^ transient duration or magnitude that might be provoked by small molecules or disease-causing mutations, we performed a series of experiments with well-annotated modifiers of cardiac physiology. To ensure changes in Ca^2+^ kinetics can be observed, we used the reverse-rate dependence phenomenon that accompanies increasing frequency of electrical stimulation. All genetically encoded probes respond to increased pacing frequencies (0.5, 1, and 2 Hz) with increased speed of binding and release as the Ca^2+^ transient duration shortens (Online Figure VI). Because the lowest pacing frequency produces the longest Ca^2+^ transient, and maximizes experimental duration, we performed subsequent experiments at 0.5 Hz.

### Small Molecule Effects on Cytoplasmic and Myofilament Ca^2+^ Dynamics

**Figure 4.**
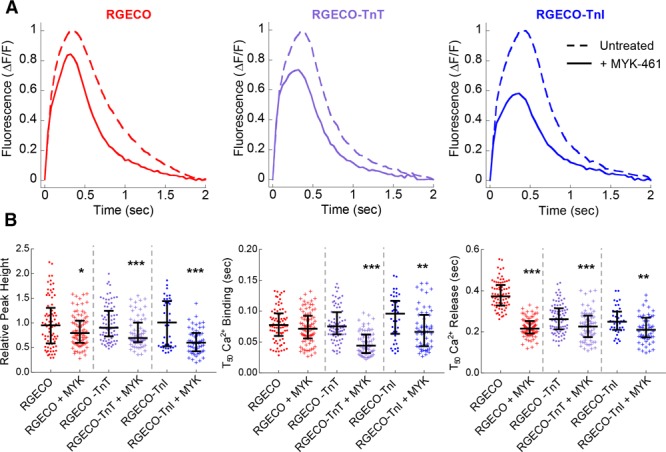
**The effects of MYK-461 on Ca^2^^+^ transient measurements in Guinea pig cardiomyocytes (GPCMs) with RGECO, RGECO-TnT (Troponin T), or RGECO-TnI (Troponin I**). Averaged Ca^2+^ transients of 0.5 Hz paced GPCMs transduced with RGECO, RGECO-TnT, or RGECO-TnI were used to compare the effects of 250 nmol/L MYK-461 (**A**). Each comparison is made in paired experiments between drug treated (solid lines) and DMSO control treated (dashed lines) for RGECO infected (red), RGECO-TnT infected (purple), and RGECO-TnI infected (blue) cells. Dot plots for all extracted parameters are plotted in (**B**; n=43–82 cells from n=3 isolations). Lines are median average and error bars are interquartile range, **P<0.01 and ***P<0.001 using an unpaired Mann-Whitney test comparing untreated to treated cells.

**Figure 5.**
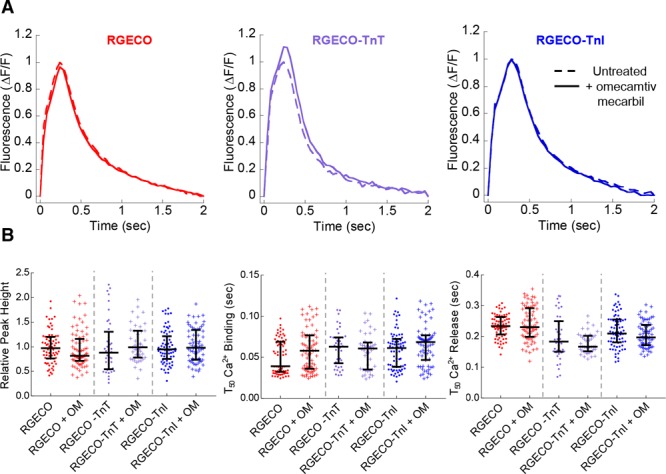
**The effects of omecamtiv mecarbil on Ca**^2^**^**+**^ transient measurements in Guinea pig cardiomyocytes (GPCMs) with RGECO, RGECO-TnT (Troponin T), or RGECO-TnI (Troponin I**). Averaged Ca^2+^ transients of 0.5 Hz paced GPCMs transduced with RGECO, RGECO-TnT, or RGECO-TnI were used to compare the effects 200 nmol/L omecamtiv mecarbil (**A**). Each comparison is made in paired experiments between drug treated (solid lines) and DMSO control treated (dashed lines) for RGECO infected (red), RGECO-TnT infected (purple), and RGECO-TnI infected (blue) cells. Dot plots for all extracted parameters are plotted in (**B**; n=42–75 cells from n=3 isolations). Lines are median average and error bars are interquartile range, all groups were not significant using an unpaired Mann-Whitney test comparing untreated to treated cells.

**Figure 6.**
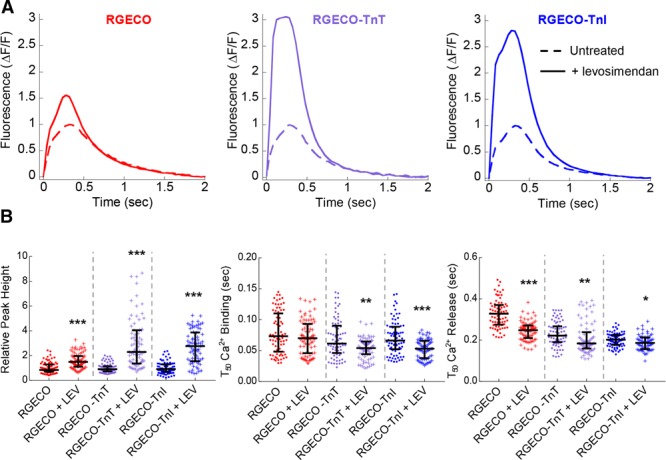
**The effects of levosimendan on Ca^2+^ transient measurements in Guinea pig cardiomyocytes (GPCMs) with RGECO, RGECO-TnT (Troponin T), or RGECO-TnI (Troponin I**). Averaged Ca^2+^ transients of 0.5 Hz paced GPCMs transduced with RGECO, RGECO-TnT, or RGECO-TnI was used to compare the effects 10 μmol/L levosimendan (**A**). Each comparison is made in paired experiments between drug treated (solid lines) and DMSO control treated (dashed lines) for RGECO infected (red), RGECO-TnT infected (purple), and RGECO-TnI infected (blue) cells. Dot plots for all extracted parameters are plotted in (**B**; n=71–83 cells from n=3 isolations). Lines are median average and error bars are interquartile range, *P<0.05, **P<0.01, and ***P<0.001 using an unpaired Mann-Whitney test comparing untreated to treated cells.

To investigate the ability of RGECO-TnI/RGECO-TnT to detect localized changes in Ca^2+^ compared with an unrestricted sensor we used 3 small molecule modulators of myofilament contractility. MYK-461 was selected as a myosin ATPase inhibitor (Figure 4). Omecamtiv mecarbil, which increases the interaction between actin and myosin independently of Ca^2+^ was selected as a myosin activator (Figure 5), and finally, the inodilator levosimendan which stabilizes the Ca^2+^ bound form of TnC activating the myofilament (Figure 6). In agreement with previous observations^48^ omecamtiv mecarbil did not change any aspect of the Ca^2+^ transient with any probe (Figure 5). By contrast, peak amplitudes increase in the presence of levosimendan and reduce in the presence of MYK-461. The myofilament-restricted indicators report greater changes in peak amplitude ratio compared with the unrestricted control. The relative reductions in fluorescence conferred by MYK-461 were −9.5±3.8%, −21.4±4.3%, and −42.7±4.1% for RGECO, RGECO-TnT, and RGECO-TnI, respectively. Analogous increases in fluorescent amplitude following levosimendan treatment were +47.5±6.6%, +198.8±23.8%, and +170.8±16.5% for RGECO, RGECO-TnT, and RGECO-TnI, respectively. Additionally, the myofilament localized RGECO reveals changes to the rate of Ca^2+^ binding and release in the presence of both MYK-461 and levosimendan contrasting with the results obtained with the cytoplasmic probe (Figures 4 and 6). Both myofilament restricted indicators in response to 250 nmol/L MYK-461 and 10 μmol/L levosimendan reduce the T_50_On times (RGECO-TnT, −0.031±0.002 s, −0.008±0.002 s; RGECO-TnI, −0.019±0.004 s to −0.018±0.002 s for MYK-461, and levosimendan, respectively) while RGECO detects no change. Interestingly, the unrestricted RGECO reports greater changes to T_50_Off compared with the restricted sensors (Figures 4 and 6), which might reflect hierarchical Ca^2+^ reuptake. Importantly, neither compound affects the intrinsic Ca^2+^ sensor function of RGECO-TnT (Online Figure VII).

### Cytoplasmic and Myofilament Localized Ca^2+^ Dynamics in HCM

Small molecule action in intact cells may include both on- and off-target components. Therefore, we explored whether the targeted RGECO strategy identified alterations in the calcium transient driven by HCM causing mutations, which should principally work through the myofilament. Specifically, previous contractility data suggest that peak amplitude should increase, and also phase shift by ≈100 ms, however, these have been undetectable with fura-2.^8^ Recombinant adenovirus expressing RGECO-TnT was used to study the effects of WT cTnI or cTnI R145G expression; conversely, adenovirus expressing RGECO-TnI was used to observe the effects of WT cTnT or cTnT R92Q in GPCMs. The mutually exclusive expression of HCM mutant protein and sensor conjugate allows changes in Ca^2+^ flux to be attributed to the mutation independently of the consequence of the mutation itself.

Previously, we showed a mutant transgene expression approach models autosomal dominant human HCM (≈54% for cTnT R92Q and ≈49% for cTnI R145^10^) and produces hypercontractility as a cellular manifestation of disease phenotype. Here, as we cotransduced separate adenoviruses containing the disease gene and the complementary Ca^2+^ indicator, adjustments to expression levels of singly versus doubly transduced cells required optimization of the multiplicities of infection, with subsequent Western blot analysis to ensure doubly transduced cells had equivalent FLAG-tagged protein expression to singly transduced cells^10^ (Online Figure VIII). Immunolocalization experiments using DsRed and FLAG-tag antibodies to detect RGECO and HCM mutant troponin show no alterations to protein localization or sarcomeric structure as previously described^10^ (Online Figure IX).

**Figure 7.**
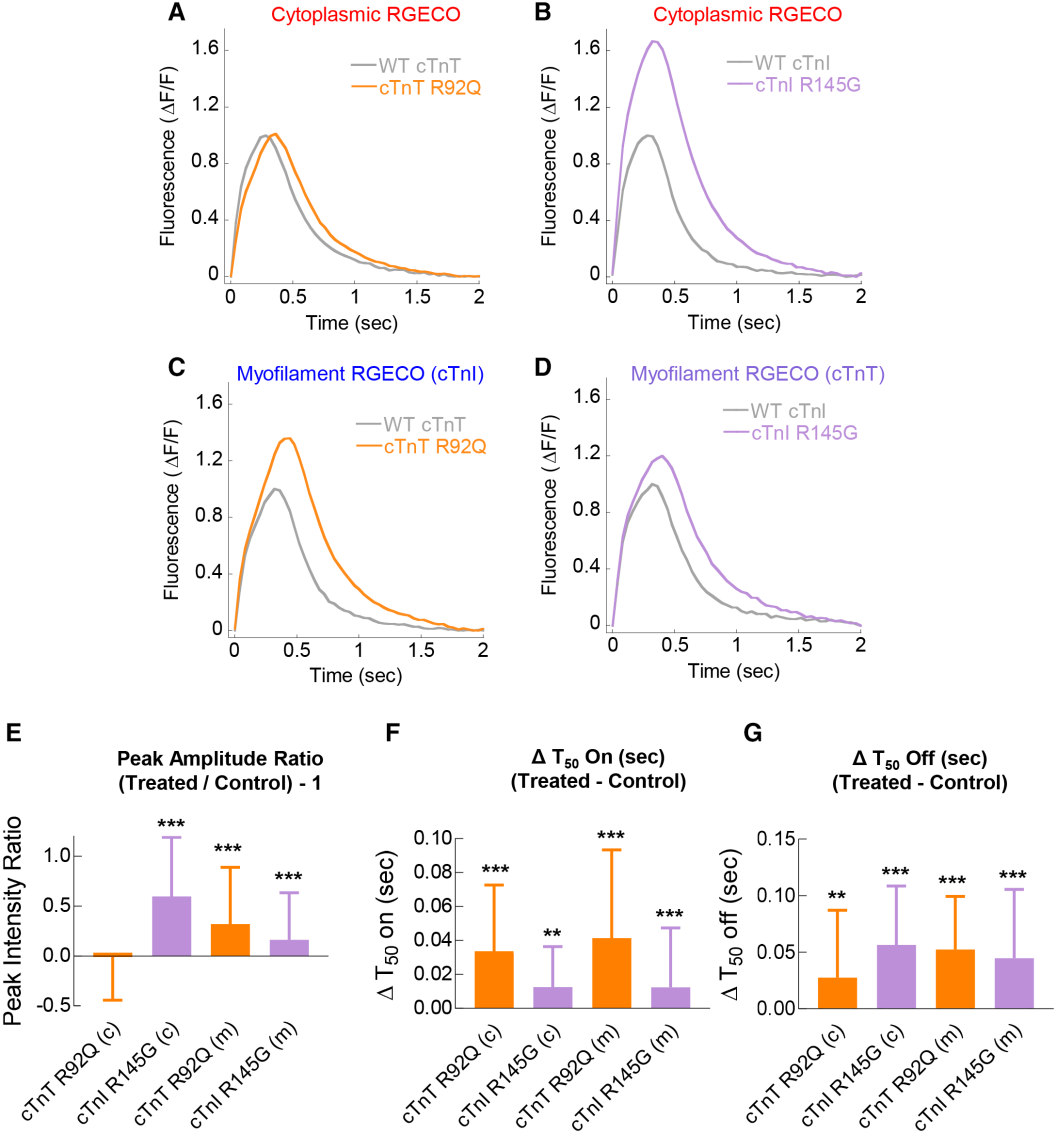
**Cytoplasmic and myofilament localized Ca^2+^ transients with adenovirally transduced cTnT R92Q and cTnI R145G.** Averaged Ca^2+^ transients of 0.5 Hz paced Guinea pig cardiomyocytes (GPCMs) transduced with RGECO (**A** and **B**) or RGECO-TnI (Troponin I)/RGECO-TnT (Troponin T; **C** and **D**) was used to compare Ca^2+^ transient effects between GPCMs transduced with either WT cTnT/cTnT R92Q (**A** and **C**), or WT cTnI/cTnI R145G (**B** and **D**). Peak amplitude ratio (**E**) and Δvalues for time to 50% binding (On; **F**) and 50% release (Off; **G**) are plotted including significance and labeled cytoplasmic (c) and myofilament (m; n=94–124 cells from n=3 isolations). Error bars are SD, **P<0.01 and ***P<0.001 using a Mann-Whitney test comparing wild-type (WT) troponin to mutant troponin transduced cells.

At the myofilament, compared with the WT control, both cTnT R92Q and cTnI R145G exert similar effects. The peak amplitude is delayed and elevated combined with slower T_50_ release times indicative of HCM causing thin filament mutations promoting more Ca^2+^ at the myofilament for longer intervals. Conversely, the whole-cell indicator reports differential changes in peak systolic Ca^2+^ only for cTnI R145G and no change in systolic Ca^2+^ for cTnT R92Q (Figure 7A through 7E; Online Table IV). The delay in time of peak Ca^2+^ correlates with previously published sarcomere length traces showing a delayed peak contraction time, which are overlooked by chemical dyes.^10^ All indicator combinations detected increased T_50_ binding in both cTnT R92Q and cTnI R145G (Figure 7F; Online Table IV) and T_50_ release predicted (Online Table IV) from previous work measuring the whole-cell Ca^2+^ dynamics.^10^

## Discussion

Here, we show that a genetically encoded Ca^2+^ sensor, RGECO, can be targeted to the myofilament without perturbing contractility, or indicator function. This allows direct Ca^2+^ measurement in a key compartment of the cardiomyocyte historically studied indirectly. The availability of 2 targeted sensors, which are almost functionally equivalent, should facilitate studies of calcium handling at the myofilament in response to drugs or disease-causing mutation.

GECIs applied to GPCMs reveal findings that contrast with calcium dyes. For example, GECIs show the myosin ATPase inhibitor MYK-461 alters Ca^2+^ binding, release and signal amplitude, none of which were previously described in chemical dye studies.^30^ Furthermore, myofilament targeting enhances the observed effect size when cardiomyocytes are treated with levosimendan. The mechanism of levosimendan action is debated, with a direct Ca^2+^ sensitizing effect meditated through TnC, a cAMP-mediated effect through phosphodiesterase III inhibition,^49^ and mitochondrial potassium channel activation^50^ all reported. Although our data do not define the relative contributions, or the intermediates in the mode of action, they do show potent elevation of myofilament Ca^2+^ is combined with an increase in total cell Ca^2+^. The whole-cell consequences are undesirable, and possibly attributable to off-target effects, as the R92Q-TnT mutant demonstrates that increased myofilament Ca^2+^ need not translate into increased whole-cell Ca^2+^. Similarly, omecamtiv mecarbil^48^ alters contraction but does not change Ca^2+^ at the whole cell, or myofilament level in this model.

The myofilament-restricted Ca^2+^ indicators reveal both cTnT R92Q and cTnI R145G HCM causing variants increase myofilament peak Ca^2+^ and delay release. Therefore, the increased myofilament Ca^2+^ affinity common to both mutations^8^ directly correlates with increased microdomain [Ca^2+^]. This is compatible with previous work showing the same mutations increased myofilament Ca^2+^ buffering and altered [Ca^2+^] inferred by a rise in diastolic fura-2 fluorescence.^10^ The RGECO-TnI/TnT data contrast with results from the unrestricted RGECO indicator; where systolic peak intensity differences are observed only for cTnI R145G (a large increase) but not cTnT R92Q suggesting distinct consequences of these mutations. Because prior work excluded other factors such as fractional Ca^2+^ release from the sarcoplasmic reticulum,^10^ the differences appear determined by what happens at the myofilament. This is mechanistically plausible as the cTnI R145G mutation directly increases Ca^2+^ binding to the regulatory site (I) of cTnC, whereas cTnT R92Q is structurally distant from the Ca^2+^ binding sites in the troponin complex and sensitizes myofilament Ca^2+^ binding via alterations to cooperative communication.^8^ Furthermore, previous studies show the cTnI R145G mutation has a greater effect on Ca^2+^ buffering than TnT R92Q (TnT R92Q ΔK_d_=582 nmol/L, TnI R145G ΔK_d_=1082 nmol/L compared with WT).^10^ We think greater severity of the R145G mutation, which has a more pronounced contractility effect,^8^ underpins the differential observations of changes to bulk Ca^2+^ with RGECO between the 2 mutants although exactly what makes the R145G cTnI mutation more severe is unknown. Interestingly, a CRISPR engineered thick filament HCM iPS-CM (induced pluripotent stem-cell derived cardiomyocyte) model (MYH7 R453C) has raised peak systolic Ca^2+^ visualized by unrestricted RGECO.^45^ From this, we postulate that cytoplasmic Ca^2+^ increases could be a hallmark of some HCM causing mutations. This raises an important, potentially clinically significant, concept. HCM represents a common clinical end point of many different mutations. There is an unmet need to understand the shared and distinct consequences of individual mutations. Common mechanisms central to disease pathogenesis may be preferable as therapeutic targets, but they may leave residual aspects of individual mutations untreated. The impact of this is currently uncertain, but we may gain insight in to this through trials such as EXPLORER investigating MYK-461 in HCM cohorts.

Ca^2+^ overload specifically at the myofilament is thought to activate hypertrophic signaling pathways as a component of the HCM disease mechanism,^13,51,52^ however, measuring this directly in living cells has remained elusive. This is the first study to not only show this occurs, but also that the phase shift in contractility is paralleled by a shift in the Ca^2+^ transient using any of the 3 protein indicators tested. These indicators simplify and accelerate the process of studying Ca^2+^ dynamics at the myofilament without the need for multiple inferred observations of total and free Ca^2+^ using simultaneous patch clamping and chemical Ca^2+^ dyes.^10,11,20^ Although the proteins used to detect Ca^2+^ are physically bulky and may have unmeasured effects on the intricate structure of the myofilament, or myofilament Ca^2+^ buffering capacity, contractile impairment is not seen in the GPCM transient expression model; this contrasts with the chemical dyes typically used to study Ca^2+^.^10,53^ A limitation of the RGECO indicators is their intensiometric nature, which precludes robust calibration allowing an observed signal to be converted to [Ca^2+^]. However, GECIs reveal changes in Ca^2+^ amplitude and kinetics that are overlooked with dye-based probes. This opens a new investigative pathway in understanding this important microdomain within contractile cells which should be generally applicable to conditions affecting cardiac contractility and Ca^2+^ handling. They improve the fidelity of screening tools for small molecule evaluation in the heart.

## Sources of Funding

This work was supported by the British Heart Foundation (Programme grant RG/12/16/29939 to H. Watkins and C.S. Redwood) and the British Heart Foundation Centre of Research Excellence (Oxford, RE/13/1/30181). Y.-F. Chang was supported by a pump-priming grant from the British Heart Foundation Centre of Excellence award to Oxford University (RE/08/004/23915 and RE/13/1/30181). M.J. Daniels is funded by the Wellcome Trust (WT098519MA) and the Japan Society for the Promotion of Science (JSPS) international joint research promotion program at Osaka University.

## Disclosures

None.

## Supplementary Material

## Novelty and Significance

### What Is Known?

- Hypertrophic cardiomyopathy mutations and small molecule modulators of contractility alter how the cardiomyocyte handles calcium.
- Historical methods to measure calcium at the myofilament are indirect because the chemical calcium dyes commonly used to visualize calcium are not possible to confine to subcellular regions.
- An additional limitation of using chemical calcium dyes in this context is their reported attenuation of contractility.
