# Supplementary material for: Measurement of Myofilament-Localized Calcium Dynamics in Adult Cardiomyocytes and the Effect of Hypertrophic Cardiomyopathy Mutations
Source: Circ Res. 2019 Apr 11;124(8):1228–39. doi: 10.1161/CIRCRESAHA.118.314600 (PMC6485313; doi:10.1161/CIRCRESAHA.118.314600)
Supplement: Supplementary file 2 [file res-124-1228-s002.pdf]

SUPPLEMENTAL MATERIAL

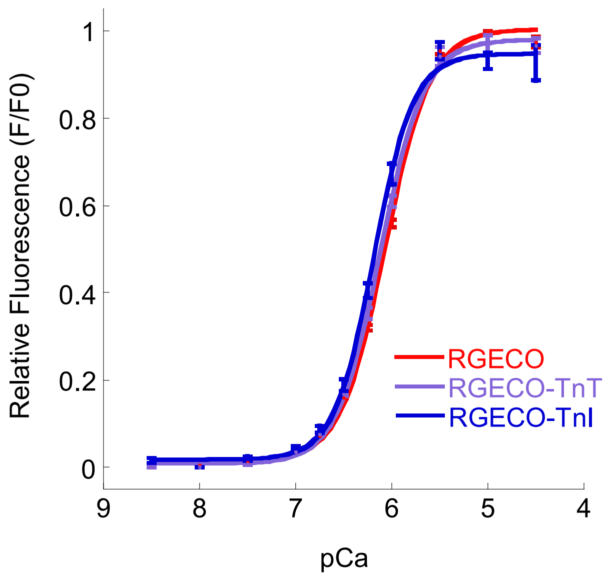

Online Figure I. Ca^{2+} binding affinity plot of RGECO, RGECO-TnT and RGECO-TnI. The steady state Ca^{2+} binding affinity of purified recombinant RGECO (red lines) RGECO-TnT (purple lines) and RGECO-TnI (blue lines) was assessed by analysis of the fluorescence/pCa relationship at 37 °C, 1.3 mM $MgCl_2$ and pH 7.3 (n=4).

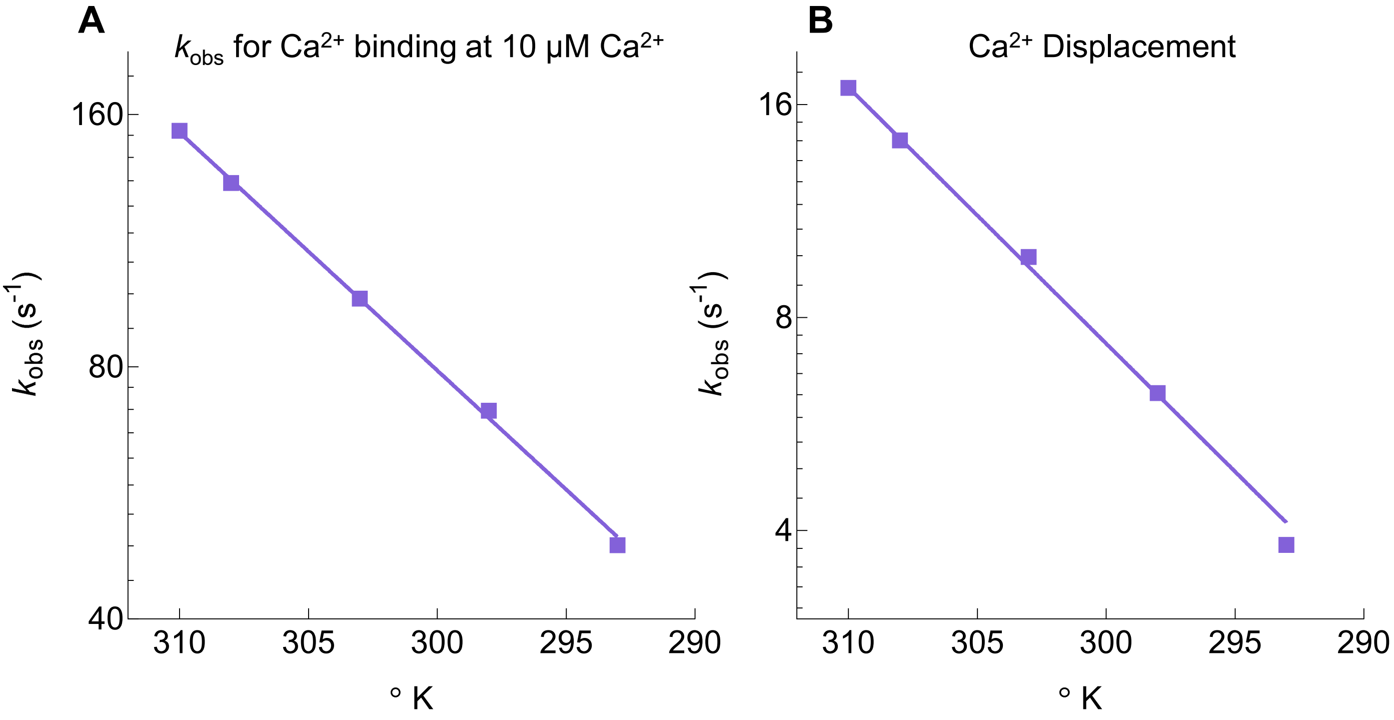

C

	k_{off}/s^{-1}		k_{on}/s^{-1}	
**Temp (°C)**	**25**	**37**	**25**	**37**
**RGECO-TnT**	6.3	16.9	70.9	167.0

Online Figure II. Kinetic determination of k_{on} and k_{off} for RGECO-TnT by stopped flow. Arrhenius plots of the observed rate constant of Ca^{2+} binding **(A)** and Ca^{2+} release rate constant **(B)** as determined by stopped flow measurement of 0.125 μM purified RGECO-TnT protein. Data for room (25 °C) and body (37 °C) temperature are shown in **(C).**

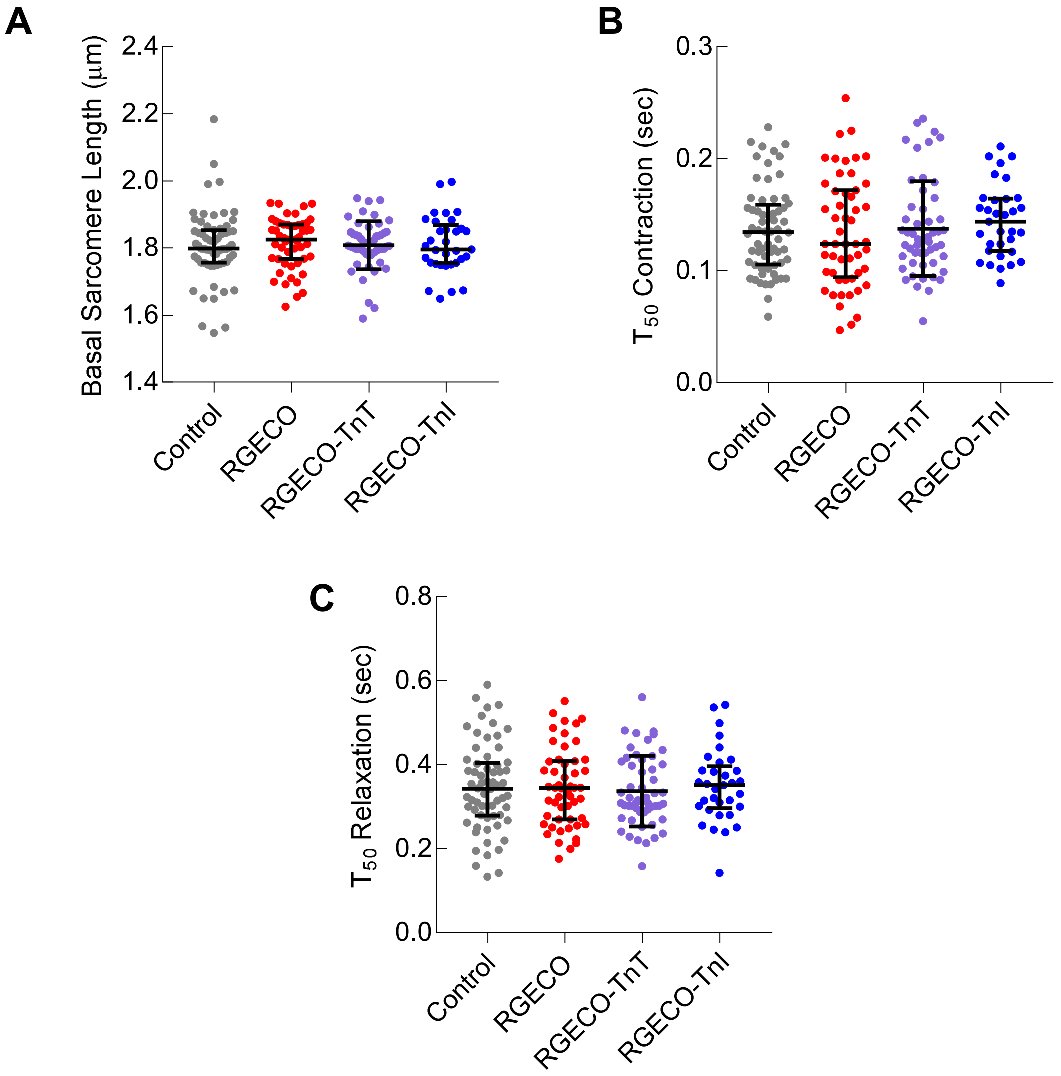

Online Figure III. Contractile properties of RGECO, RGECO-TnT and RGECO-TnI. Contractile parameters during electrical paced (0.5 Hz) of isolated adult cardiomyocytes transduced and either RGECO, RGECO-TnT or RGECO-TnI showing no change in basal sarcomere length (**A**), time to 50% contraction (**B**) or time to 50% relaxation (**C**) (n=33-64 cells from n=3 isolations).

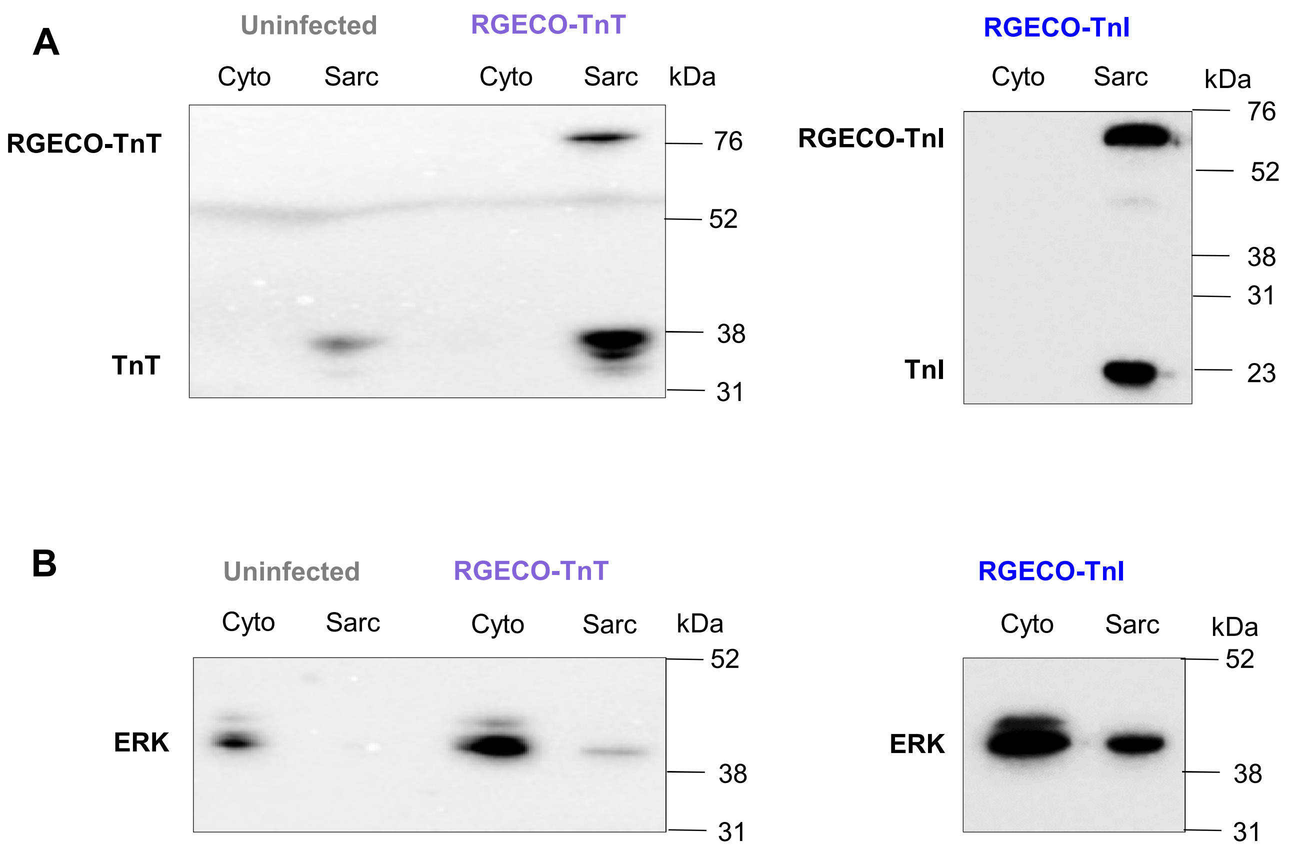

Online Figure IV. Subcellular fractionation of GPCMs expressing RGECO-TnT and RGECO-TnI. The relative subcellular incorporation of RGECO-TnT or RGECO-TnI was assessed by subcellular fractionation of GPCMs 48 hours after adenoviral infection. Western blots using anti-cTnT or anti-cTnI gave an endogenous band and a conjugate band on the sarcomeric fraction (sarc) **(A)**. Re-probing with the predominantly cytoplasmic marker anti-ERK showed that subcellular fractionation was of high fidelity **(B)**.

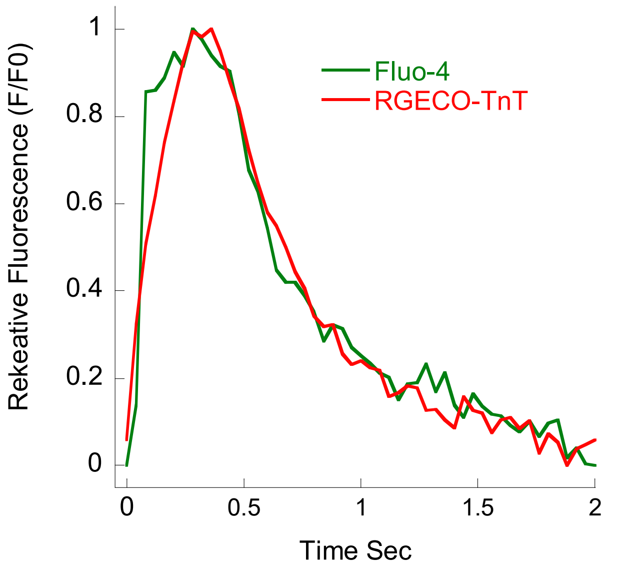

	Fluo-4	RGECO-TnT
**T_{50} on (sec)**	0.059±0.003	0.088±0.004 *
**T_{50} off (sec)**	0.418±0.026	0.316±0.038 *

Online Figure V. The on and off rates of Ca^{2+} transients measured with Fluo-4 are significantly different to RGECO-TnT in simultaneous measurements if dye and sensor in the same cell. Fluorescence measurements taken from cells loaded with 0.5 µmol/L Fluo-4 and expressing RGECO-TnT were measured at 488 nm and 595 nm respectively. Emitted transients were from the same cell and therefore enabled direct pairwise comparison of T_{50} on and T_{50} off rates (n=82 cells from n=3 isolations). Data presented as mean ± SEM, * = $p<0.05$, using Wilcoxon paired t-test.

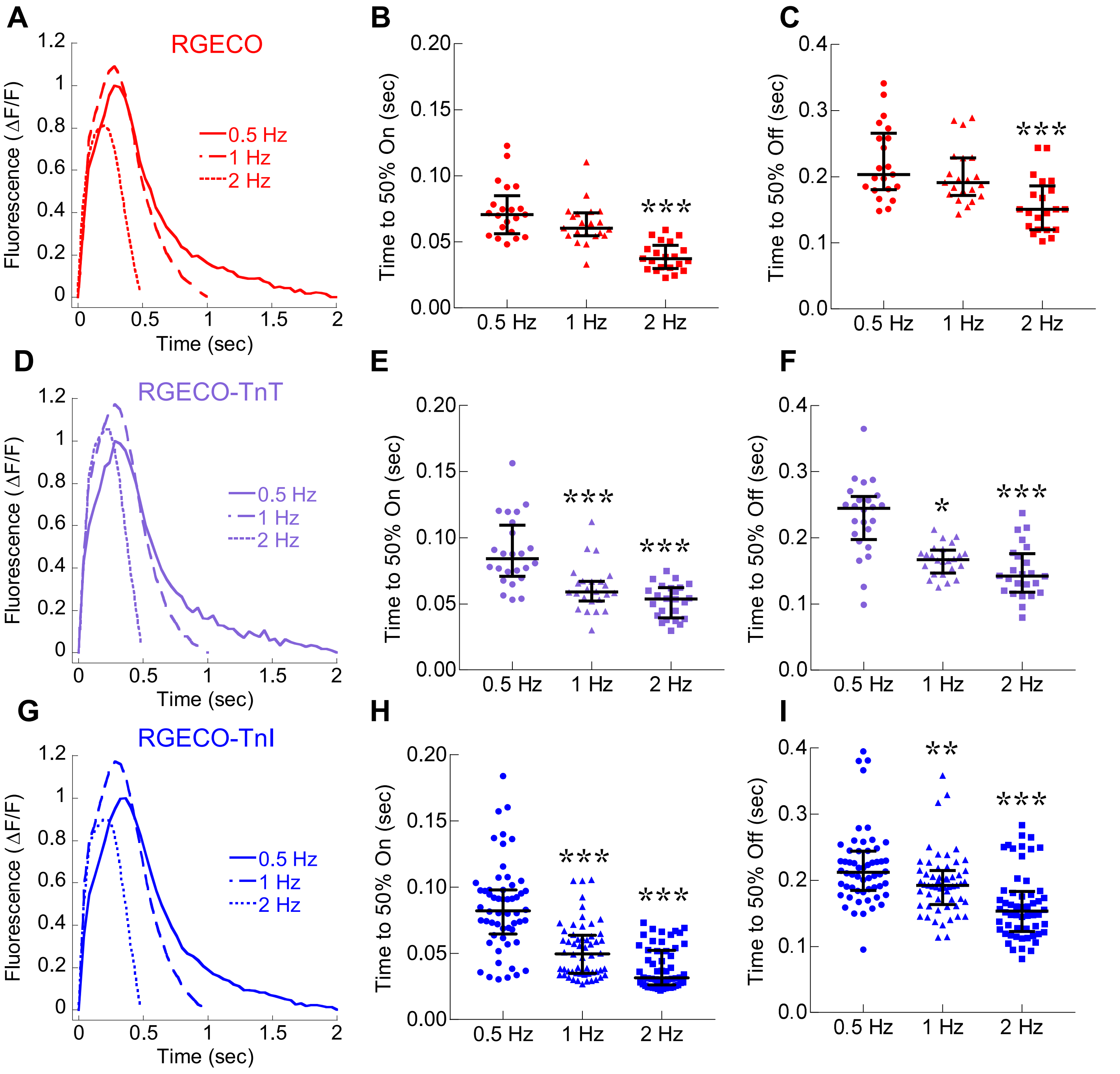

Online Figure VI. RGECO, RGECO-TnT and RGECO-TnI Ca^{2+} transients show reverse rate dependence in times to 50% on and 50% off in response to increased pacing frequency in adult GPCMs. Averaged Ca^{2+} transients of electrically paced isolated adult cardiomyocytes was used to compare pacing frequencies of 0.5 Hz, 1.0 Hz and 2 Hz using either RGECO **(A)**, RGECO-TnT **(D)** or RGECO-TnI **(G).** Adjacent dot plots show distributions of time to 50% binding, and 50% release rates across the pacing range. Shortening of both parameters is apparent for all indicators (RGECO **(B** and **C)** (n=21 cells from n=2 isolations), RGECO-TnT **(E** and **F)** (n=24 cells from n=2 isolations), and RGECO-TnI **(H** and **I)** (n=56 cells from n=3 isolations)). Lines give the median average and error bars are ± interquartile range * = p<0.05, ** = p<0.01 and *** = p<0.001 using one way ANOVA.

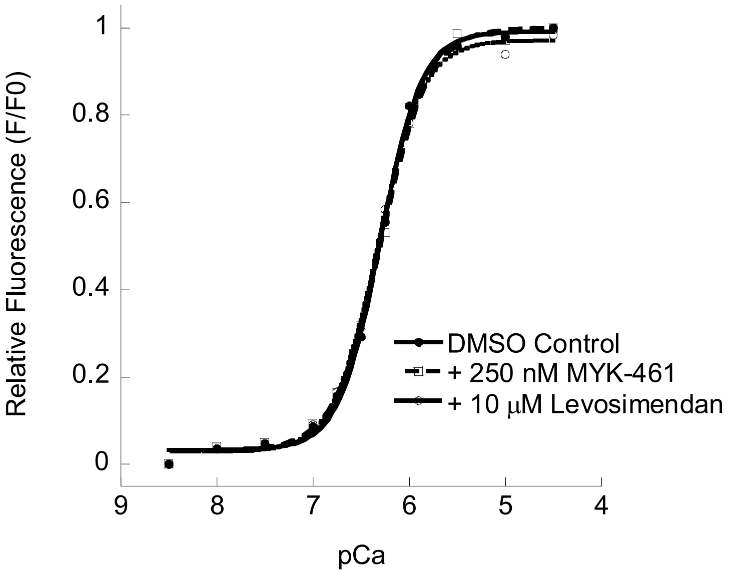

	K_d	p
**Control**	502 ± 23.0 nM	
**MYK461**	522 ± 26.9 nM	0.602
**Levosimendan**	475 ± 19.3 nM	0.471

Online Figure VII. MYK-461 and levosimendan had no direct effect on the function of the RGECO-TnT. Fluorescence : pCa relationship in the presence of 250 nmol/L MYK-461 and 10 μmol/L levosimendan was used to calculate the K_d values tabulated beneath (n=4). Significance values (p) were calculated using one way ANOVA.

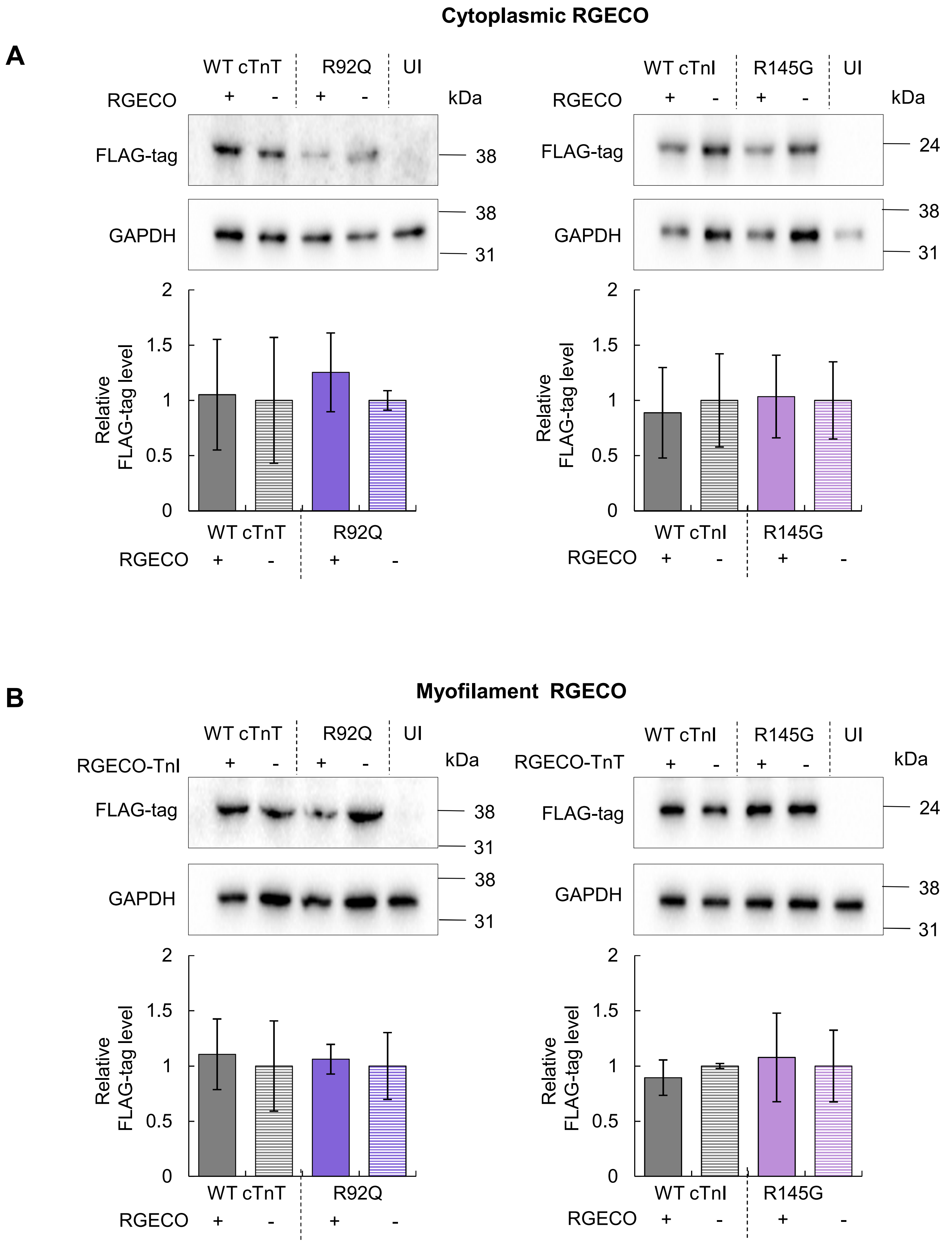

Online Figure VIII. Effect of dual transduction versus single transduction of adenoviruses on FLAG-tag expression. Western blot analysis of GPCMs transduced with WT cTnT, cTnT R92Q, WT cTnI, or cTnI R145G ± RGECO **(A)** or RGECO-TnI/RGECO-TnT **(B)**, or uninfected control (UI). Showing no significant effect on FLAG-tag expression between dual transduction with the Ca^{2+} sensor and single transduction without the Ca^{2+} sensor, n=5.

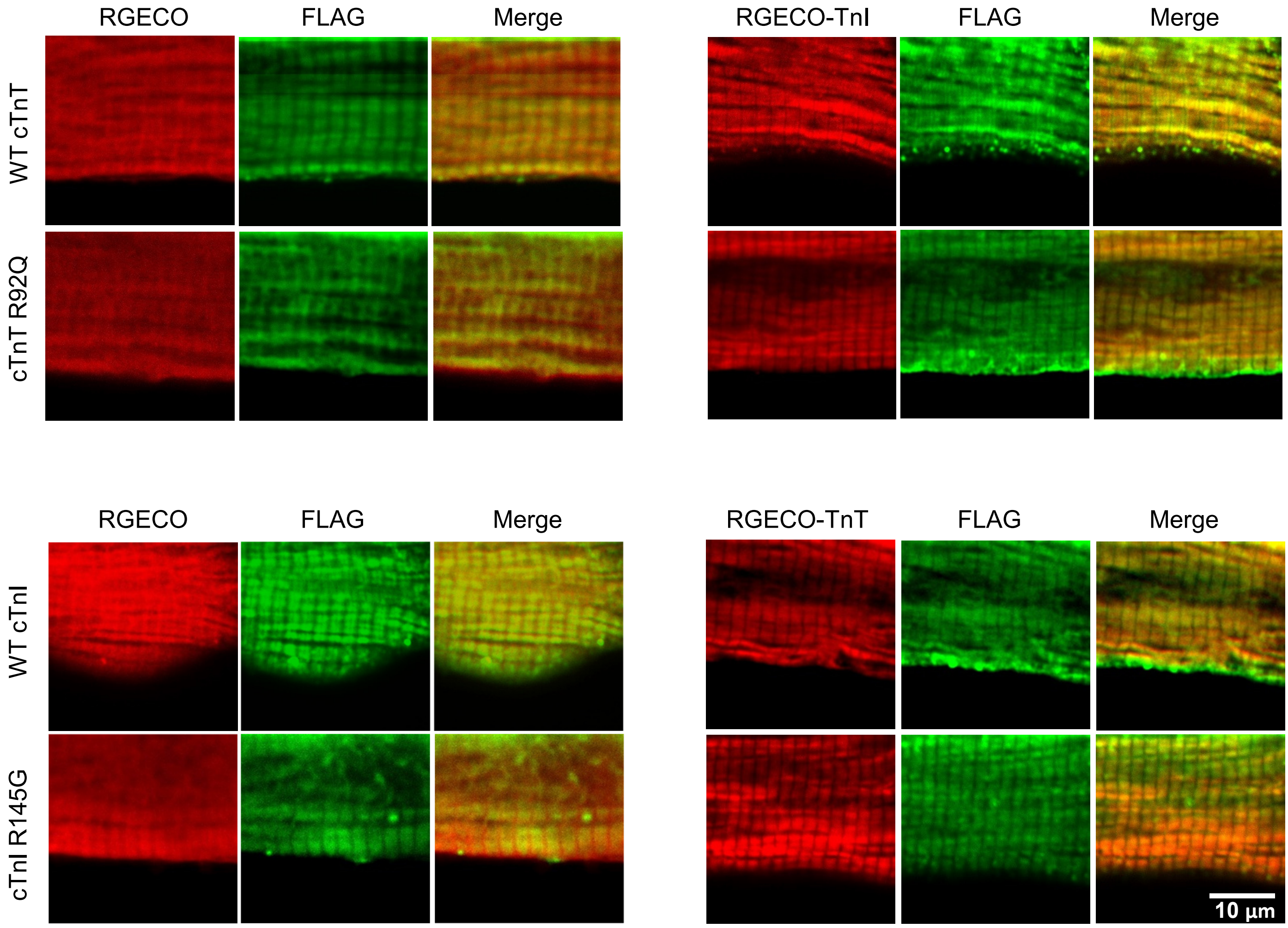

Online Figure IX. Effect of dual transduction on the localisation of FLAG-tag expressed protein. Immunofluorescence analysis of GPCMs transduced with WT cTnT, cTnT R92Q, WT cTnI, or cTnI R145G ± RGECO/RGECO-TnI/RGECO-TnT was performed using anti-FLAG tag (green) and anti-DsRed antibodies. No significant alterations to the I band localisation of FLAG-tag protein was observed between dual transduction with the Ca^{2+} sensor and previously described single transduction without the Ca^{2+} sensor.

RGECO-TnT DNA sequence

ATGGTAGACTCATCACGTCGTAAGTGGAATAAGGCAGGTCACGCAGTCAGAGCTATAGGTCGGCTGAGCTCACCCGTGGTT
TCCGAGCGGATGTACCCCGAGGACGGCGCCCTCAAGAGCGAGATCAAGAAGGGGCTGAGGCTGAAGGACGGCGGCCACTAC
GCCGCCGAGGTCAAGACCACCTACAAGGCCAAGAAGCCCGTGCAGCTGCCCGGCGCCTACATCGTAGACATCAAGTTGGAC
ATCGTGTCCCACAACGAGGACTACACCATCGTGGAACAGTGCGAACGCGCCGAGGGCCGCCACTCCACCGGCGGCATGGAC
GAGCTATACAAGGGAGGTACAGGCGGGAGTCTGGTGAGCAAGGGCGAGGAGGATAACATGGCCATCATCAAGGAGTTCATG
CGCTTCAAGGTGCACATGGAGGGCTCCGTGAACGGCCACGAGTTCGAGATCGAGGGCGAGGGCGAGGGCCGCCCCTACGAG
GCCTTTCAGACCGCTAAGCTGAAGGTGACCAAGGGTGGCCCCCTGCCCTTCGCCTGGGACATCCTGTCCCCTCAGTTCATG
TACGGCTCCAAGGCCTACATTAAGCACCCAGCCGACATCCCCGACTACTTCAAGCTGTCCTTCCCCGAGGGCTTCAGGTGG
GAGCGCGTGATGAACTTCGAGGACGGCGGCATTATTCACGTTAACCAGGACTCCTCCCTGCAGGACGGCGTATTCATCTAC
AAGGTGAAGCTGCGCGGCACCAACTTCCCCCCCGACGGCCCCGTAATGCAGAAGAAGACCATGGGCTGGGAGGCTACGCGT
GACCAACTGACTGAGGAGCAGATCGCAGAATTTAAAGAGGCTTTCTCCCTATTTGACAAGGACGGGGATGGGACGATAACA
ACCAAGGAGCTGGGGACGGTGATGCGGTCTCTGGGGCAGAACCCCACAGAAGCAGAGCTGCAGGACATGATCAATGAAGTA
GATGCCGACGGTGACGGCACATTCGACTTCCCTGAGTTCCTGACGATGATGGCAAGAAAAATGAATGACACAGACAGTGAA
GAGGAAATTAGAGAAGCGTTCCGCGTGTTTGATAAGGACGGCAATGGCTACATCGGCGCAGCAGAGCTTCGCCACGTGATG
ACAGACCTTGGAGAGAAGTTAACAGATGAGGAGGTTGATGAAATGATCAGGGTAGCAGACATCGATGGGGATGGTCAGGTA
AACTACGAAGAGTTTGTCCAAATGATGACAGCGAAGGGATCCATGTCTGACATAGAAGAGGTGGTGGAAGAGTACGAGGAG
GAGGAGCAGGAAGAAGCAGCTGTTGAAGAGCAGGAGGAGGCAGCGGAAGAGGATGCTGAAGCAGAGGCTGAGACCGAGGAG
ACCAGGGCAGAAGAAGATGAAGAAGAAGAGGAAGCAAAGGAGGCTGAAGATGGCCCAATGGAGGAGTCCAAACCAAAGCCC
AGGTCGTTCATGCCCAACTTGGTGCCTCCCAAGATCCCCGATGGAGAGAGAGTGGACTTTGATGACATCCACCGGAAGCGC
ATGGAGAAGGACCTGAATGAGTTGCAGGCGCTGATTGAGGCTCACTTTGAGAACAGGAAGAAAGAGGAGGAGGAGCTCGTT
TCTCTCAAAGACAGGATCGAGAGACGTCGGGCAGAGCGGGCCGAGCAGCAGCGCATCCGGAATGAGCGGGAGAAGGAGCGG
CAGAACCGCCTGGCTGAAGAGAGGGCTCGACGAGAGGAGGAGGAGAACAGGAGGAAGGCTGAGGATGAGGCCCGGAAGAAG
AAGGCTTTGTCCAACATGATGCATTTTGGGGGTTACATCCAGAAGCAGGCCCAGACAGAGCGGAAAAGTGGGAAGAGGCAG
ACTGAGCGGGAAAAGAAGAAGAAGATTCTGGCTGAGAGGAGGAAGGTGCTGGCCATTGACCACCTGAATGAAGATCAGCTG
AGGGAGAAGGCCAAGGAGCTGTGGCAGAGCATCTATAACTTGGAGGCAGAGAAGTTCGACCTGCAGGAGAAGTTCAAGCAG
CAGAAATATGAGATCAATGTTCTCCGAAACAGGATCAACGATAACCAGAAAGTCTCCAAGACCCGCGGGAAGGCTAAAGTC
ACCGGGCGCTGGAAATAG

RGECO-TnT amino acid sequence

MVDSSRRKWNKAGHAVRAIGRLSSPVVSERMYPEDGALKSEIKKGLRLKDGGHYAAEVKTTYKAKKPVQLPGAYIVDIKLD
IVSHNEDYTIVEQCERAEGRHSTGGMDELYKGGTGGSLVSKGEEDNMAIIKEFMRFKVHMEGSVNGHEFEIEGEGEGRPYE
AFQTAKLKVTKGGPLPFAWDILSPQFMYGSKAYIKHPADIPDYFKLSFPEGFRWERVMNFEDGGIIHVNQDSSLQDGVFIY
KVKLRGTNFPPDGPVMQKKTMGWEATRDQLTEEQIAEFKEAFSLFDKDGDGTITTKELGTVMRSLGQNPTEAELQDMINEV
DADGDGTFDFPEFLTMMARKMNDTDSEEEIREAFRVFDKDGNGYIGAAELRHVMTDLGEKLTDEEVDEMIRVADIDGDGQV
NYEEFVQMMTAKGSMSDIEEVVEEYEEEEQEEAAVEEQEEAAEEDAEAEAETEETRAEEDEEEEEAKEAEDGPMEESKPKP
RSFMPNLVPPKIPDGERVDFDDIHRKRMEKDLNELQALIEAHFENRKKEEEELVSLKDRIERRRAERAEQQRIRNEREKER
QNRLAEERARREEEENRRKAEDEARKKKALSNMMHFGGYIQKQAQTERKSGKRQTEREKKKKILAERRKVLAIDHLNEDQL
REKAKELWQSIYNLEAEKFDLQEKFKQQKYEINVLRNRINDNQKVSKTRGKAKVTGRWK.

Online Figure X. DNA and amino acid sequence of RGECO-TnT. Sequence highlighted red is RGECO, purple is human cardiac TnT and black is a BamHI cloning site which corresponds to an additional GS linker sequence in the translated amino acid.

RGECO-TnI DNA sequence

ATGGTAGACTCATCACGTCGTAAGTGGAATAAGGCAGGTCACGCAGTCAGAGCTATAGGTCGGCTGAGCTCACCCGTGGTT
TCCGAGCGGATGTACCCCGAGGACGGCGCCCTCAAGAGCGAGATCAAGAAGGGGCTGAGGCTGAAGGACGGCGGCCACTAC
GCCGCCGAGGTCAAGACCACCTACAAGGCCAAGAAGCCCGTGCAGCTGCCCGGCGCCTACATCGTAGACATCAAGTTGGAC
ATCGTGTCCCACAACGAGGACTACACCATCGTGGAACAGTGCGAACGCGCCGAGGGCCGCCACTCCACCGGCGGCATGGAC
GAGCTATACAAGGGAGGTACAGGCGGGAGTCTGGTGAGCAAGGGCGAGGAGGATAACATGGCCATCATCAAGGAGTTCATG
CGCTTCAAGGTGCACATGGAGGGCTCCGTGAACGGCCACGAGTTCGAGATCGAGGGCGAGGGCGAGGGCCGCCCCTACGAG
GCCTTTCAGACCGCTAAGCTGAAGGTGACCAAGGGTGGCCCCCTGCCCTTCGCCTGGGACATCCTGTCCCCTCAGTTCATG
TACGGCTCCAAGGCCTACATTAAGCACCCAGCCGACATCCCCGACTACTTCAAGCTGTCCTTCCCCGAGGGCTTCAGGTGG
GAGCGCGTGATGAACTTCGAGGACGGCGGCATTATTCACGTTAACCAGGACTCCTCCCTGCAGGACGGCGTATTCATCTAC
AAGGTGAAGCTGCGCGGCACCAACTTCCCCCCCGACGGCCCCGTAATGCAGAAGAAGACCATGGGCTGGGAGGCTACGCGT
GACCAACTGACTGAGGAGCAGATCGCAGAATTTAAAGAGGCTTTCTCCCTATTTGACAAGGACGGGGATGGGACGATAACA
ACCAAGGAGCTGGGGACGGTGATGCGGTCTCTGGGGCAGAACCCCACAGAAGCAGAGCTGCAGGACATGATCAATGAAGTA
GATGCCGACGGTGACGGCACATTCGACTTCCCTGAGTTCCTGACGATGATGGCAAGAAAAATGAATGACACAGACAGTGAA
GAGGAAATTAGAGAAGCGTTCCGCGTGTTTGATAAGGACGGCAATGGCTACATCGGCGCAGCAGAGCTTCGCCACGTGATG
ACAGACCTTGGAGAGAAGTTAACAGATGAGGAGGTTGATGAAATGATCAGGGTAGCAGACATCGATGGGGATGGTCAGGTA
AACTACGAAGAGTTTGTCCAAATGATGACAGCGAAGCTCGAGATGGCGGATGGGAGCAGCGATGCGGCTAGGGAACCTCGC
CCTGCACCAGCCCCAATCAGACGCCGCTCCTCCAACTACCGCGCTTATGCCACGGAGCCGCACGCCAAGAAAAAATCTAAG
ATCTCCGCTTCTAGAAAATTGCAGCTGAAGACTCTGCTGCTGCAGATTGCAAAGCAAGAGCTGGAGCGAGAGGCGGAGGAG
CGGCGCGGAGAGAAGGGGCGCGCTCTGAGCACCCGCTGCCAGCCGCTGGAGTTGACCGGGCTGGGCTTCGCGGAGCTGCAG
GACTTGTGCCGACAGCTCCACGCCCGTGTGGACAAGGTGGATGAAGAGAGATACGACATAGAGGCAAAAGTCACCAAGAAC
ATCACGGAGATTGCAGATCTGACTCAGAAGATCTTTGACCTTCGAGGCAAGTTTAAGCGGCCCACCCTGCGGAGAGTGAGG
ATCTCTGCAGATGCCATGATGCAGGCGCTGCTGGGGGCCCGGGCTAAGGAGTCCCTGGACCTGCGGGCCCACCTCAAGCAG
GTGAAGAAGGAGGACACCGAGAAGGAAAACCGGGAGGTGGGAGACTGGCGGAAGAACATCGATGCACTGAGTGGAATGGAG
GGCCGCAAGAAAAAGTTTGAGAGCTGA

RGECO-TnI amino acid sequence

MVDSSRRKWNKAGHAVRAIGRLSSPVVSERMYPEDGALKSEIKKGLRLKDGGHYAAEVKTTYKAKKPVQLPGAYIVDIKLD
IVSHNEDYTIVEQCERAEGRHSTGGMDELYKGGTGGSLVSKGEEDNMAIIKEFMRFKVHMEGSVNGHEFEIEGEGEGRPYE
AFQTAKLKVTKGGPLPFAWDILSPQFMYGSKAYIKHPADIPDYFKLSFPEGFRWERVMNFEDGGIIHVNQDSSLQDGVFIY
KVKLRGTNFPPDGPVMQKKTMGWEATRDQLTEEQIAEFKEAFSLFDKDGDGTITTKELGTVMRSLGQNPTEAELQDMINEV
DADGDGTFDFPEFLTMMARKMNDTDSEEEIREAFRVFDKDGNGYIGAAELRHVMTDLGEKLTDEEVDEMIRVADIDGDGQV
NYEEFVQMMTAKLEMADGSSDAAREPRPAPAPIRRRSSNYRAYATEPHAKKKSKISASRKLQLKTLLLQIAKQELEREAEE
RRGEKGRALSTRCQPLELTGLGFAELQDLCRQLHARVDKVDEERYDIEAKVTKNITEIADLTQKIFDLRGKFKRPTLRRVR
ISADAMMQALLGARAKESLDLRAHLKQVKKEDTEKENREVGDWRKNIDALSGMEGRKKKFES.

Online Figure XI. DNA and amino acid sequence of RGECO-TnI. Sequence highlighted red is RGECO, blue is human cardiac TnI and black is a XhoI cloning site which corresponds to an additional LE linker sequence in the translated amino acid.

	GFP-TnT	TnT-GFP	GFP-TnI	TnI-GFP	GFP-TnC	TnC-GFP
**ΔpCa_{50}**	-0.031±0.018	-0.149±0.056	0.028±0.015	-0.279±0.624	0.564±0.269	-0.369±0.125
**Δn_H**	0.980±0.691	7.308±3.703	0.505±1.085	**-2.158±0.170 ****	0.8649±0.598	-0.867±0.912
**ΔMin (sec^{-1})**	0.548±0.219	0.185±0.089	0.235±0.114	**3.096±1.105 ***	**6.551±0.969 *****	**5.382±1.874 ***
**ΔMax (sec^{-1})**	0.686±0.588	**-2.958±0.399 ***	-0.036±0.270	0.787±0.457	1.479±0.798	**1.439±0.545 ***

Online Table I. Extracted parameters from *in vitro* actin activated acto-myosin S1 ATPase assays performed to investigate myofilament function in the presence of GFP conjugates of the troponin complex. Δ values from paired experimental comparisons for pCa_{50}, n_H, maximum and minimum activity (sec^{-1}) with standard error and significance. n=5, significance values comparing unconjugated with GFP conjugated troponin are p<0.001=***, p<0.01=** and p<0.05=* using a students t-test.

	RGECO	RGECO-TnT	RGECO-TnI
K_d at 25 °C (nM)	1607	1186	1105
K_d at 37 °C (nM)	860	764	657
Quantum Yield pCa 4.5 (Φ)	0.20	0.33	0.3
Quantum Yield pCa 8.5 (Φ)	0.06	0.11	0.1
Molar Extinction coefficent pCa 4.5 (ε (mM^{-1}•cm^{-1})) (565 nm)	32.39	34.53	35.87
Molar Extinction coefficent pCa 8.5 (ε (mM^{-1}•cm^{-1})) (565 nm)	5.17	6.64	6.85
Molar Extinction coefficent pCa 4.5 (ε (mM^{-1}•cm^{-1})) (455 nm)	4.16	6.18	6.2
Molar Extinction coefficent pCa 8.5 (ε (mM^{-1}•cm^{-1})) (455 nm)	11.25	14.11	16.36
Brightness pCa 4.5 (mM^{-1}•cm^{-1})	6.48	11.30	13.58
Brightness pCa 8.5 (mM^{-1}•cm^{-1})	0.31	0.73	1.21
Intensity change ± Ca^{2+}	10.18x	10.33x	11.25x

Online Table II. Extracted parameters from fluorescence and absorbance spectra and kinetic experiments. Brightness is defined as the product of ε and Φ.

Sensor	*n*	Time to 50% Ca^{2+} Binding (sec)	Time to 50% Ca^{2+} Release (sec)
RGECO	*108*	0.058±0.003	0.308±0.012
RGECO-TnT	*55*	**0.105±0.007 *****	**0.282±0.011 ****
RGECO-TnI	*112*	**0.097±0.004 *****	**0.267±0.006 *****

Online Table III. Extracted parameters of GPCM Ca^{2+} transients transduced with RGECO, RGECO-TnT or RGECO-TnI. Extracted values from a paired comparison of GPCMs adenovirally transduced with RGECO, RGECO-TnT or RGECO-TnI. *n* = total cell number from at least 3 separate cell isolations, ± = standard error. Significance values comparing RGECO to RGECO-TnT or RGECO-TnI are $p<0.01$ = ** and $p<0.001$ = ***. RGECO-TnT verses RGECO-TnI = ns for all parameters, using a Kruskal Wallis non parametric test.

A

Sensor	Mutant	*n*	Relative RGECO Peak Intensity	Time to 50% Ca^{2+} Binding (sec)	Time to 50% Ca^{2+} Release (sec)	Time of Peak (sec)
RGECO	WT cTnT	*94*	1.00±0.041	0.066±0.002	0.219±0.006	0.288±0.007
RGECO	cTnT R92Q	*94*	0.99±0.045	**0.102±0.004 *****	**0.247±0.006 ****	**0.365±0.007 *****
RGECO	WT cTnI	*107*	1.00±0.037	0.070±0.002	0.198±0.004	0.281±0.006
RGECO	cTnI R145G	*103*	**1.60±0.058 *****	**0.083±0.002 *****	**0.255±0.005 *****	**0.345±0.008 *****

B

Sensor	Mutant	*n*	Relative RGECO Peak Intensity	Time to 50% Ca^{2+} Binding (sec)	Time to 50% Ca^{2+} Release (sec)	Time of Peak (sec)
RGECO-TnI	WT cTnT	*102*	1.00±0.039	0.087±0.003	0.194±0.004	0.333±0.007
RGECO-TnI	cTnT R92Q	*115*	**1.32±0.053 *****	**0.128±0.005 *****	**0.247±0.004 *****	**0.424±0.008 *****
RGECO-TnT	WT cTnI	*123*	1.00±0.030	0.082±0.003	0.202±0.005	0.338±0.008
RGECO-TnT	cTnI R145G	*127*	**1.16±0.042 ****	**0.094±0.003 ****	**0.247±0.005 *****	**0.391±0.010 *****

Online Table IV. Extracted parameters of GPCMs transduced with RGECO or myofilament localised RGECO and WT/HCM mutant troponin. Extracted Ca^{2+} transient values from GPCMs adenovirally transduced with RGECO (**A**) or RGECO-TnI/RGECO-TnT (**B**) and either WT cTnT, cTnT R92Q, WT cTnI, or cTnI R145G. *n* = total cell number from at least 3 separate cell isolations, ± = standard error. Significant values comparing either WT cTnT to cTnT R92Q or WT cTnI to cTnI R145G are p<0.001 = ***, or p<0.01 = ** using a Mann Whitney test.
